# Supplementary material for: Synthesis and Antimicrobial Activity of Novel 4-Hydroxy-2-quinolone Analogs
Source: Molecules. 2020 Jul 4;25(13):3059. doi: 10.3390/molecules25133059 (PMC7412474; doi:10.3390/molecules25133059)
Supplement: Supplementary file 1 [file molecules-25-03059-s001.pdf]

Supporting Information

Synthesis and Antimicrobial Activity of Novel 4-Hydroxy-2-quinolone Analogues

Thitiphong Khamkhenshorngphanuch [1,†], Kittipat Kulkraisri [2,†], Alongkorn Janjamratsaeng [2,†], Napasawan Plabutong [3], Arsa Thammahong [3], Kanitta Manadee [4], Sarisa Na Pombejra [4], and Tanatorn Khotavivattana [5,*]

[1] Department of General Education, Faculty of Science and Health Technology, Navamindradhiraj University, Bangkok 10300, Thailand; thitiphong.kha@nmu.ac.th
[2] Department of Chemistry, Faculty of Science, Chulalongkorn University, Bangkok 10330, Thailand; kittipatkulkraisri@gmail.com (K.K.); mekans89@gmail.com (A.J.)
[3] Antimicrobial Resistance and Stewardship Research Unit, Department of Microbiology, Faculty of Medicine, Chulalongkorn University, Bangkok 10330, Thailand; fah34405@gmail.com (N.P.); arsa.t@chula.ac.th (A.T.)
[4] Department of Microbiology, Faculty of Science, Chulalongkorn University, Bangkok 10330, Thailand; noonkanitta@gmail.com (K.M.); sarisa.n@chula.ac.th (S.N.)
[5] Center of Excellence in Natural Products Chemistry, Department of Chemistry, Faculty of Science, Chulalongkorn University, Bangkok 10330, Thailand
* Correspondence: tanatorn.k@chula.ac.th; Tel.: +66-2-218-7621
† These authors contributed equally to this work.

Content

1. NMR and HRMS spectra S2
   1.1 NMR spectra of **1a-1g** S2
   1.2 NMR spectra of **2a-2b** S9
   1.3 NMR and HRMS spectra of **3a-3j**, **4d** and **5d** S11
2. Antimicrobial Activity and Structure-Activity Relationship S34
   2.1 The resazurin microdilution assay to determine MICs of the synthesized quinolones against *S. aureus*. S34
   2.2 The MBC results of 3i and 3j against *S. aureus*. S37
   2.3 The resazurin microdilution assay to determine MICs of the synthesized quinolones against *E. coli*. S38

1. NMR and HRMS spectra

1.1. NMR spectra of 1a – 1g

1-Methyl-2H-benzo[d] [1,3] oxazine-2,4(1H)-dione (**1a**)

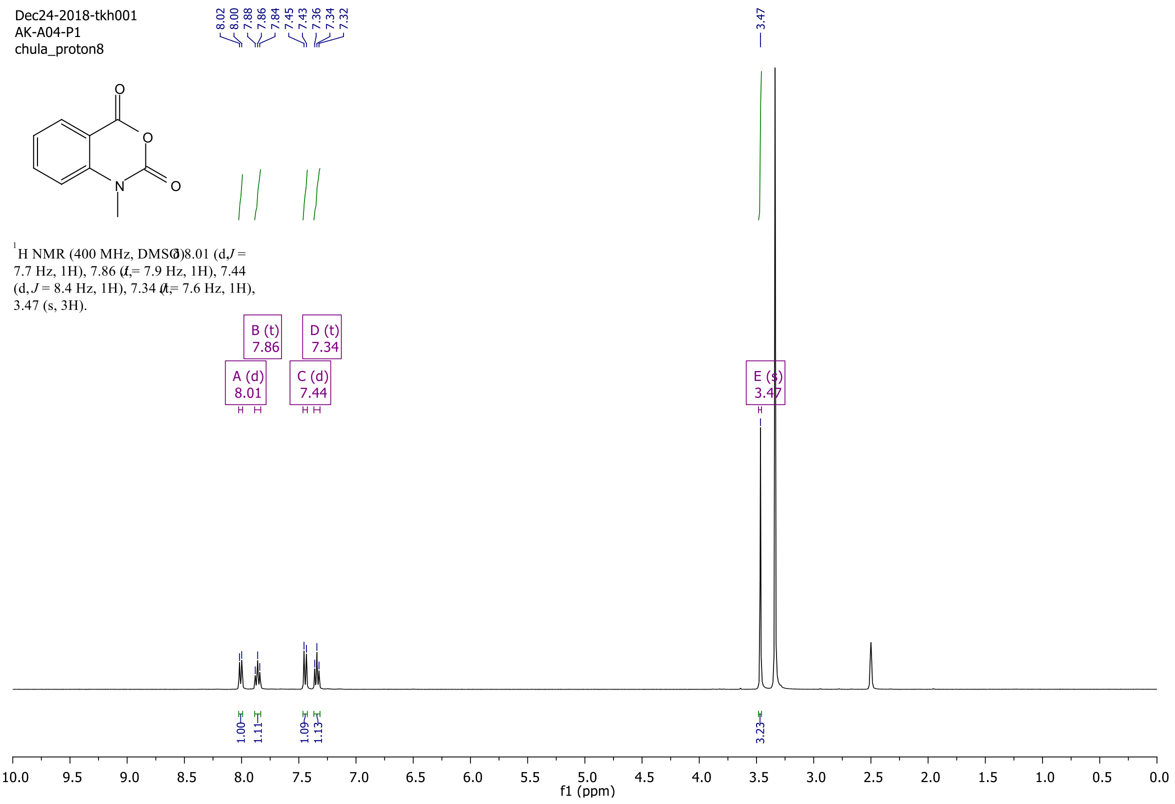

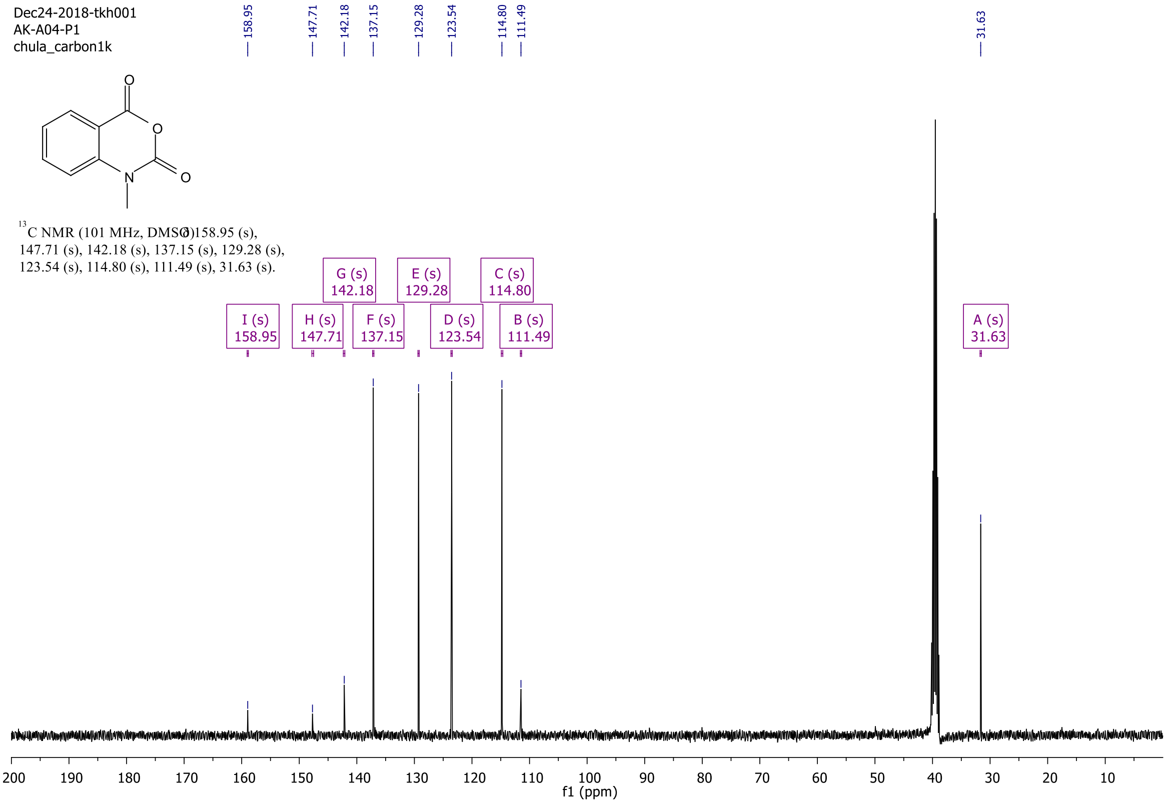

6-Methoxy-1-methyl-2H-benzo[d] [1,3] oxazine-2,4(1H)-dione (**1b**)

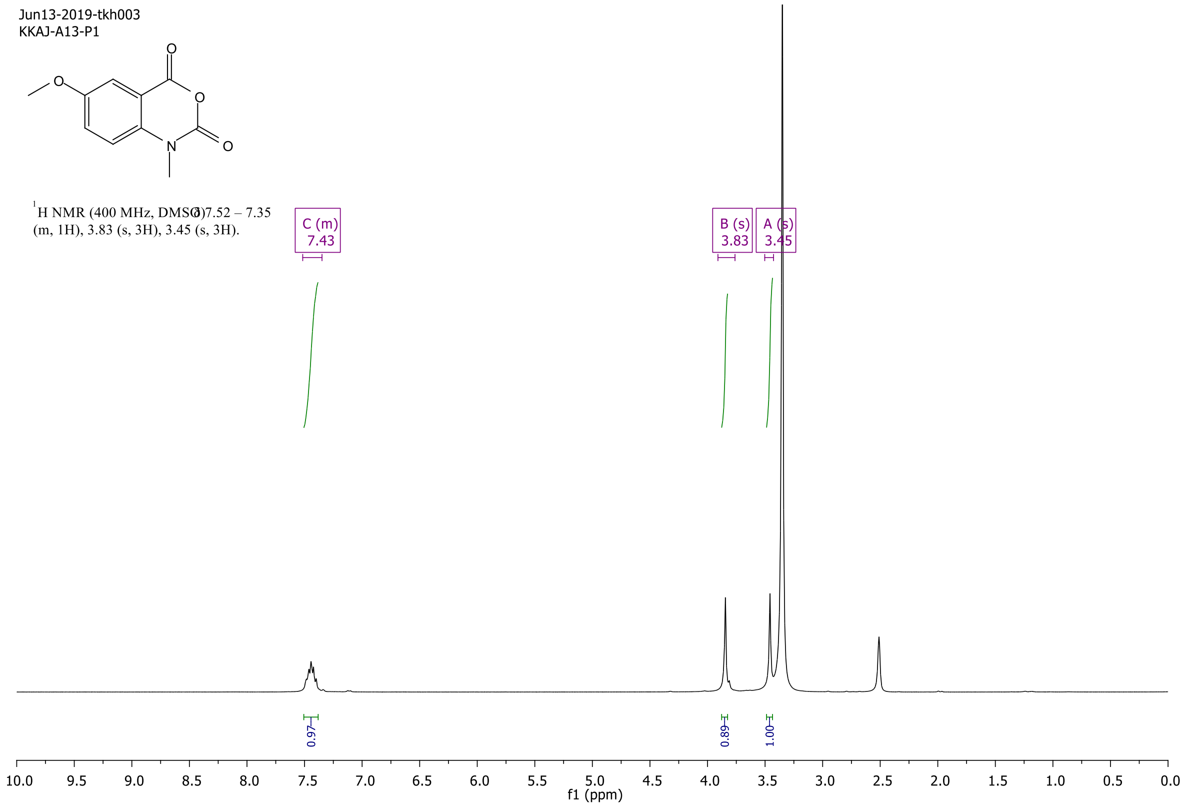

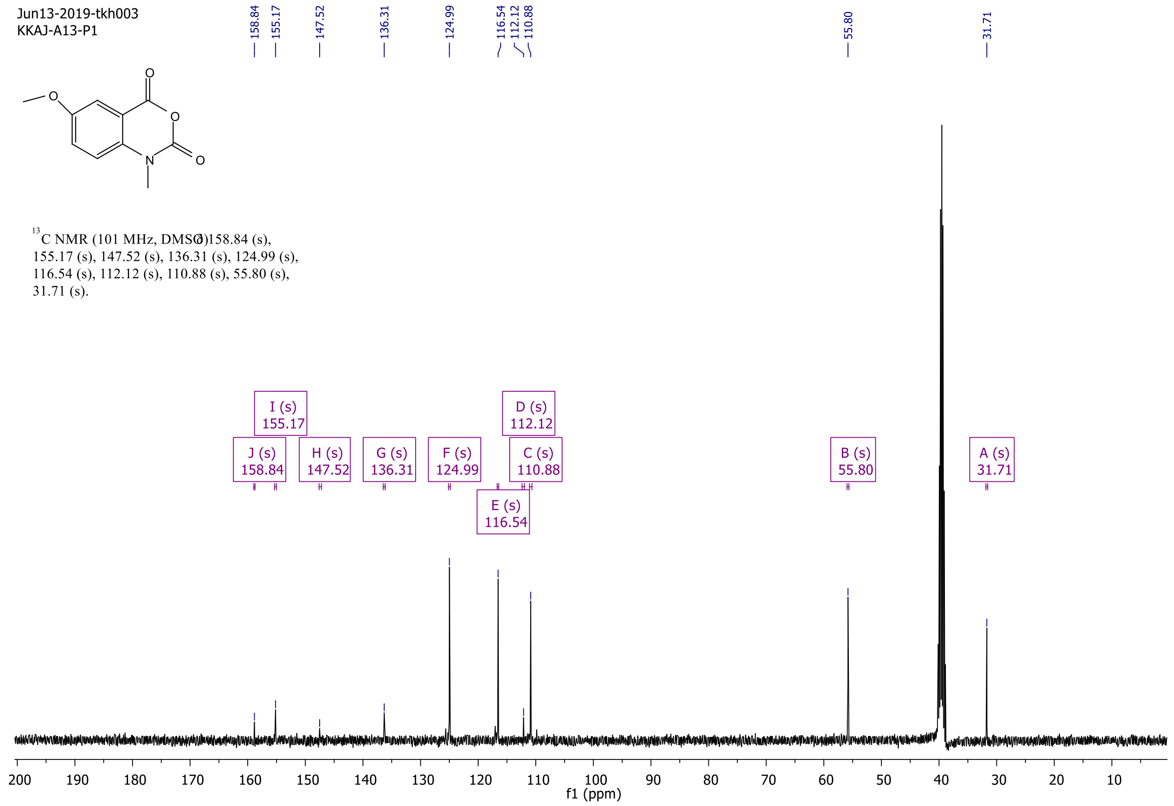

7-Chloro-1-methyl-2H-benzo[d] [1,3] oxazine-2,4 (1H)-dione (**1c**)

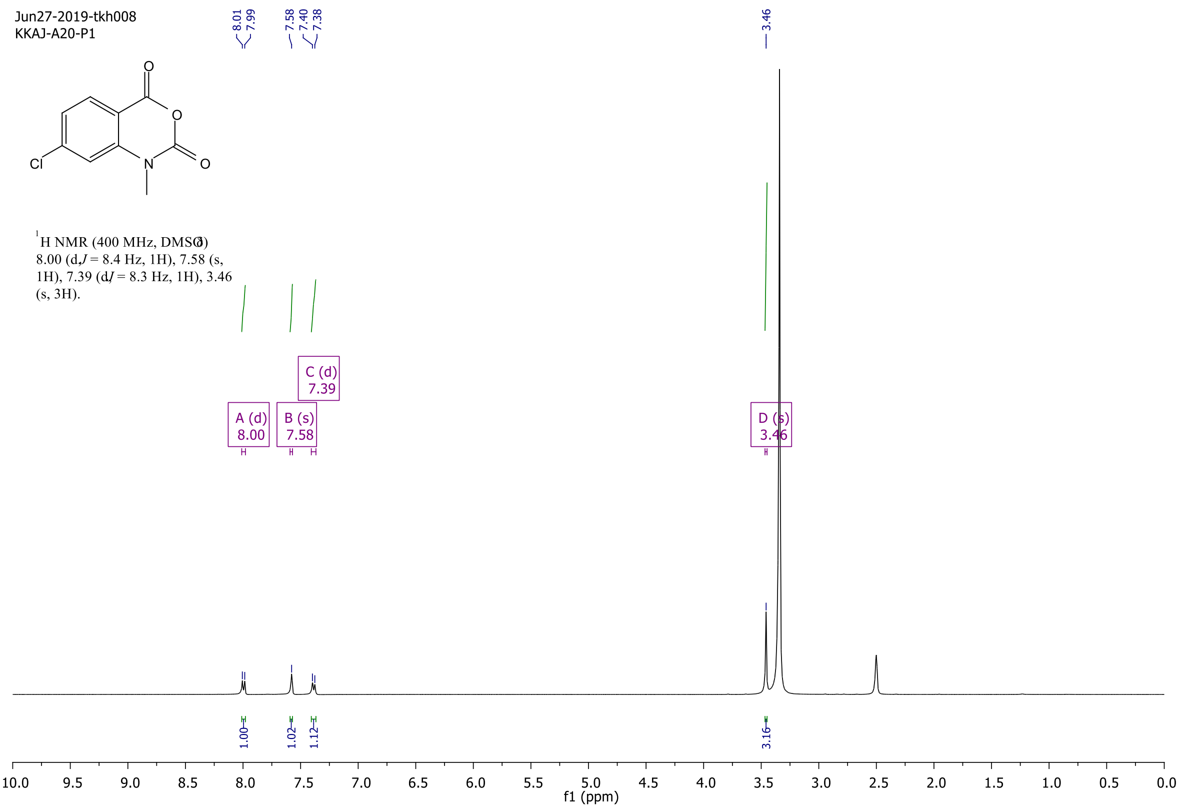

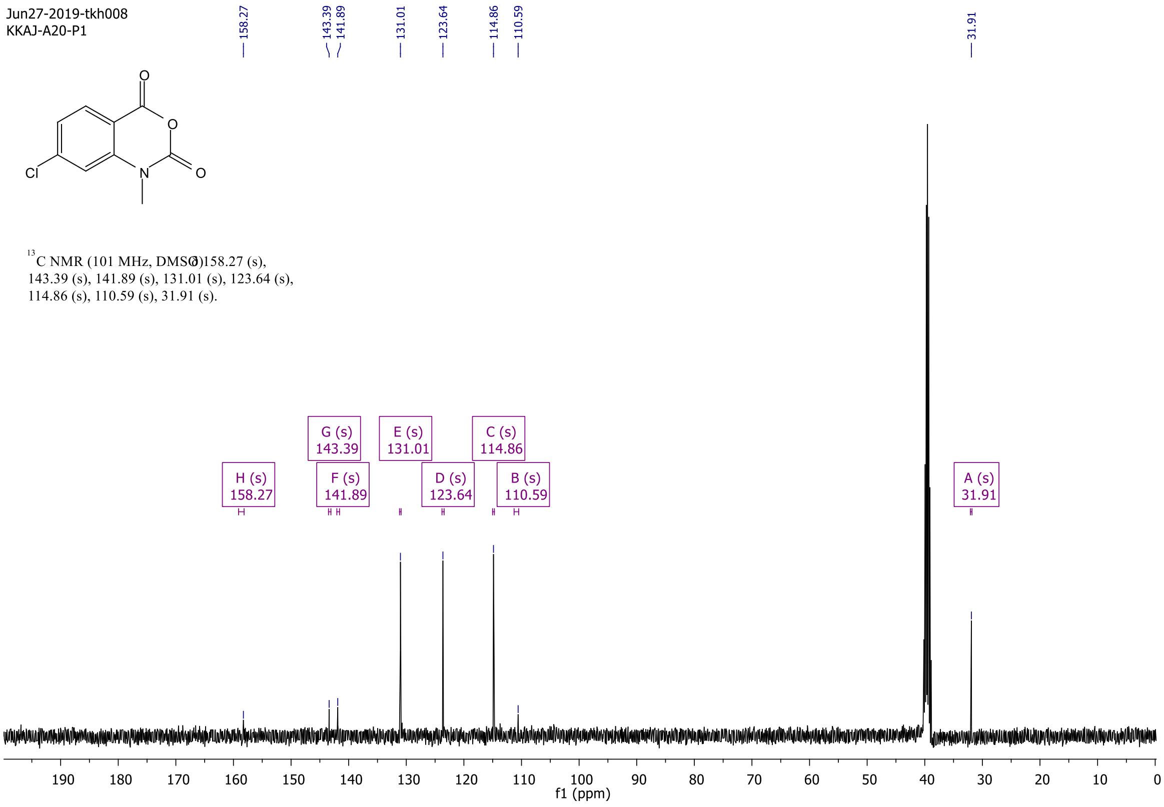

1-Methyl-7-nitro-2H-benzo[d][1,3] oxazine-2,4(1H)-dione (**1d**)

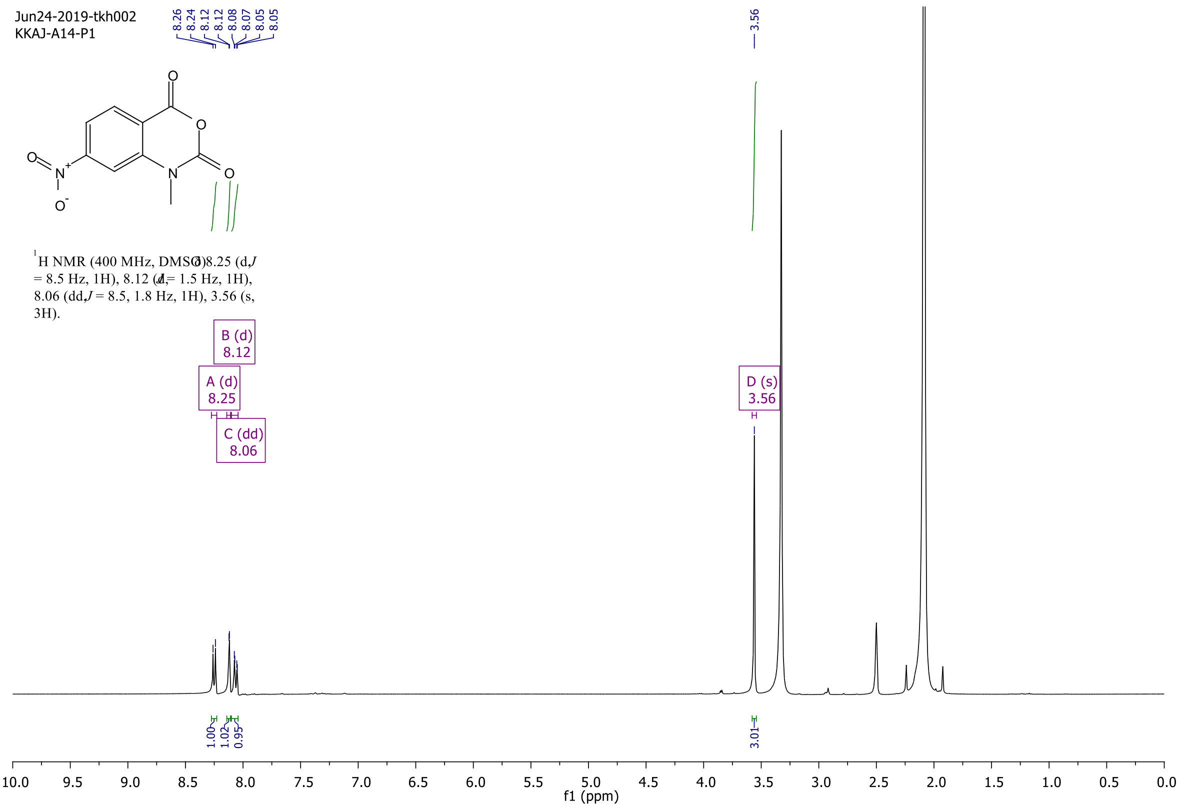

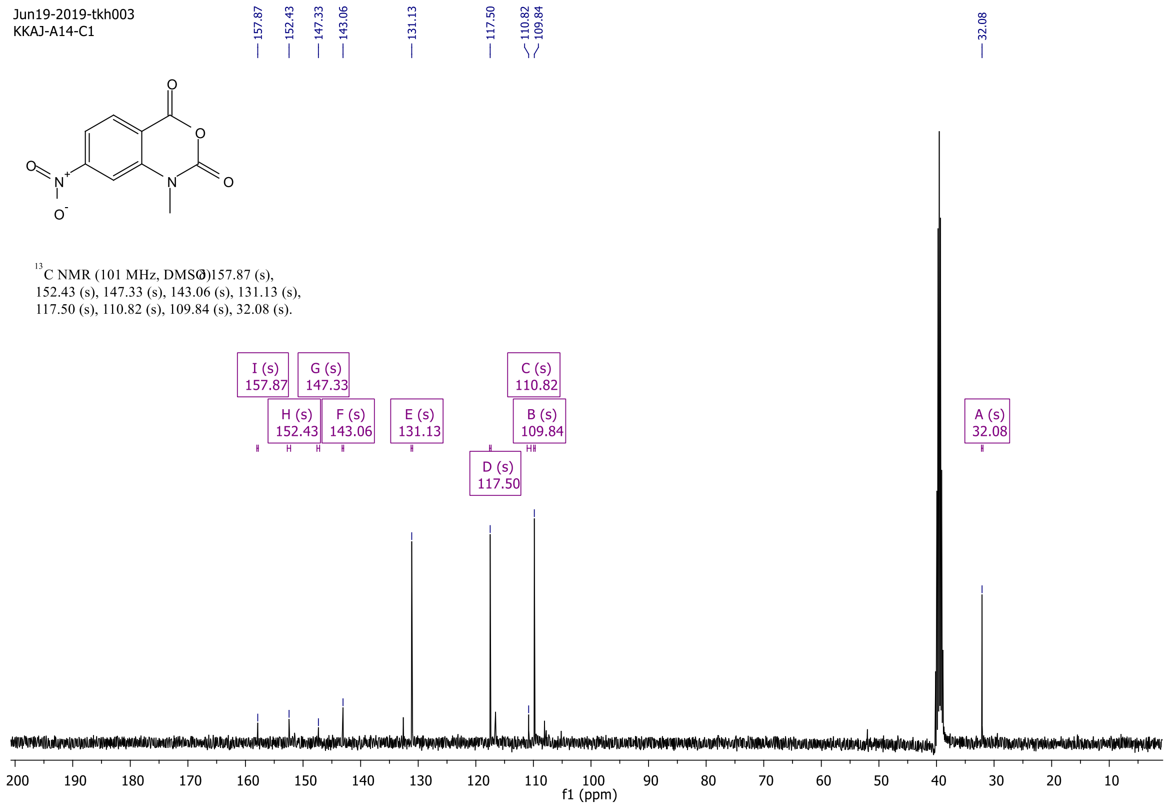

6-Chloro-1-methyl-2H-benzo[d] [1,3] oxazine-2,4(1H)-dione (**1e**)

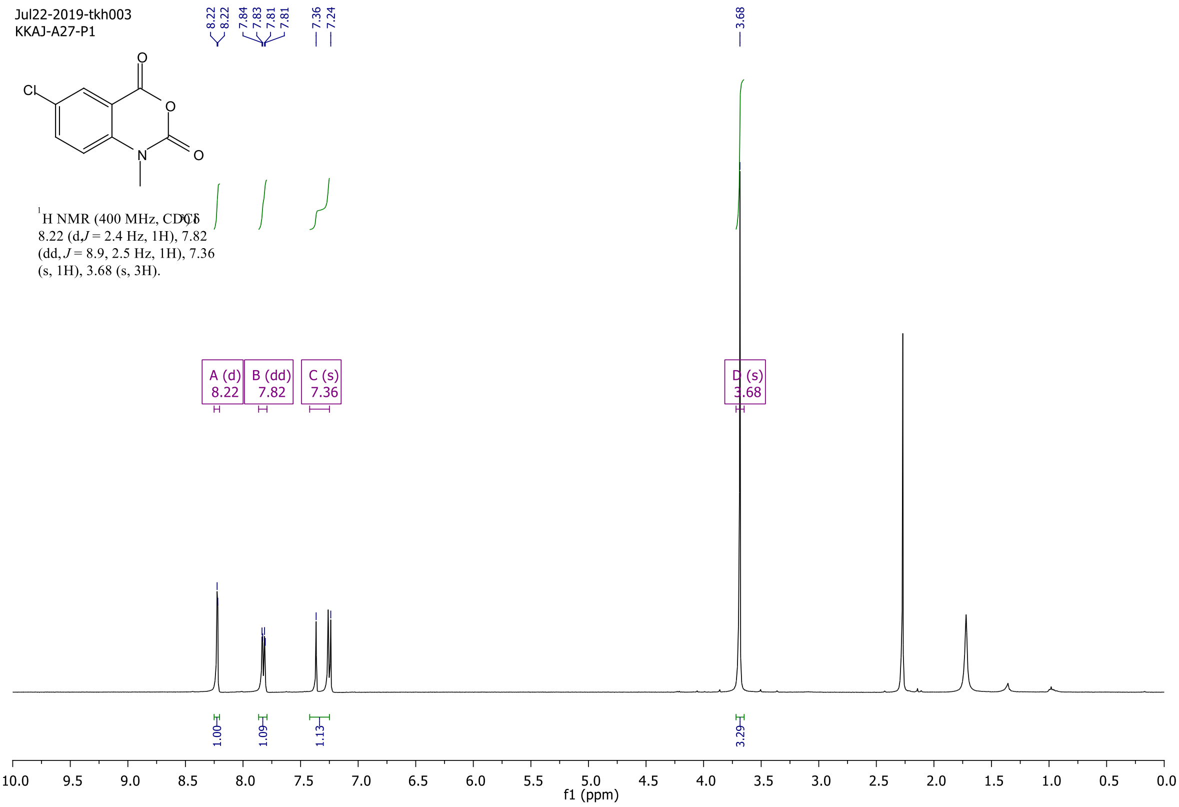

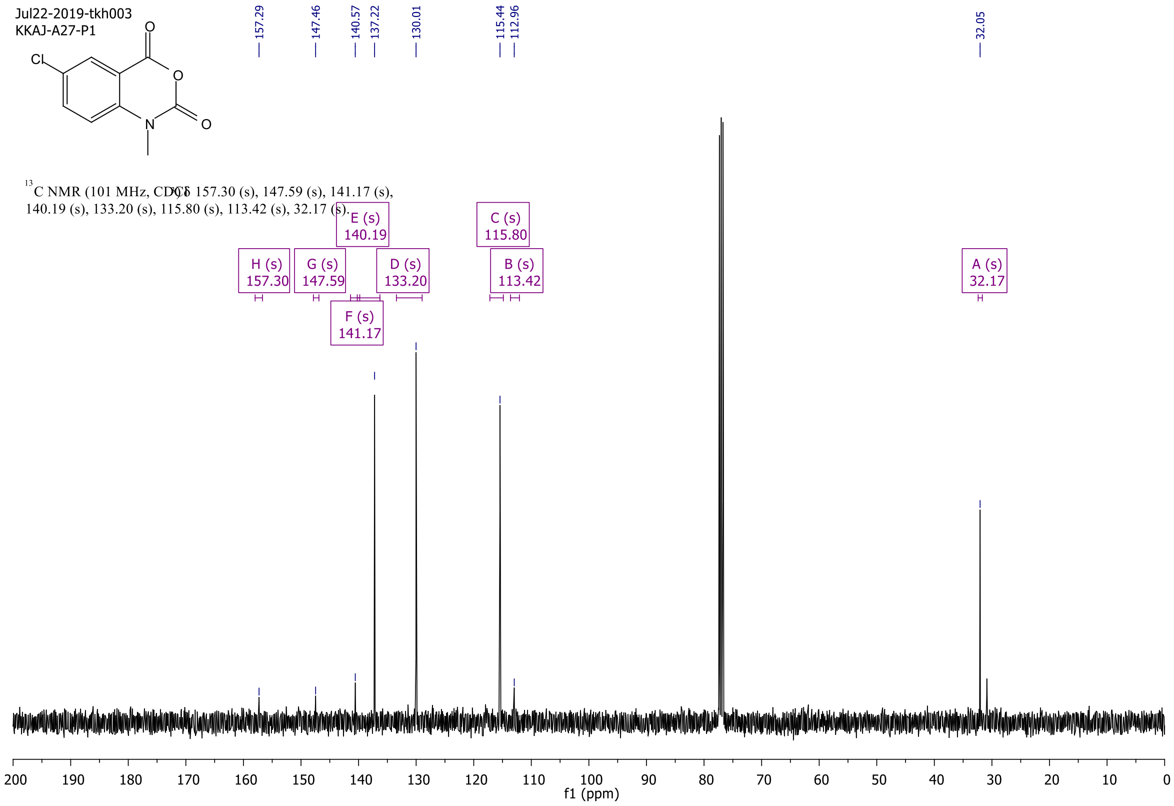

6-Bromo-1-methyl-2H-benzo[d] [1,3] oxazine-2,4(1H)-dione (**1f**)

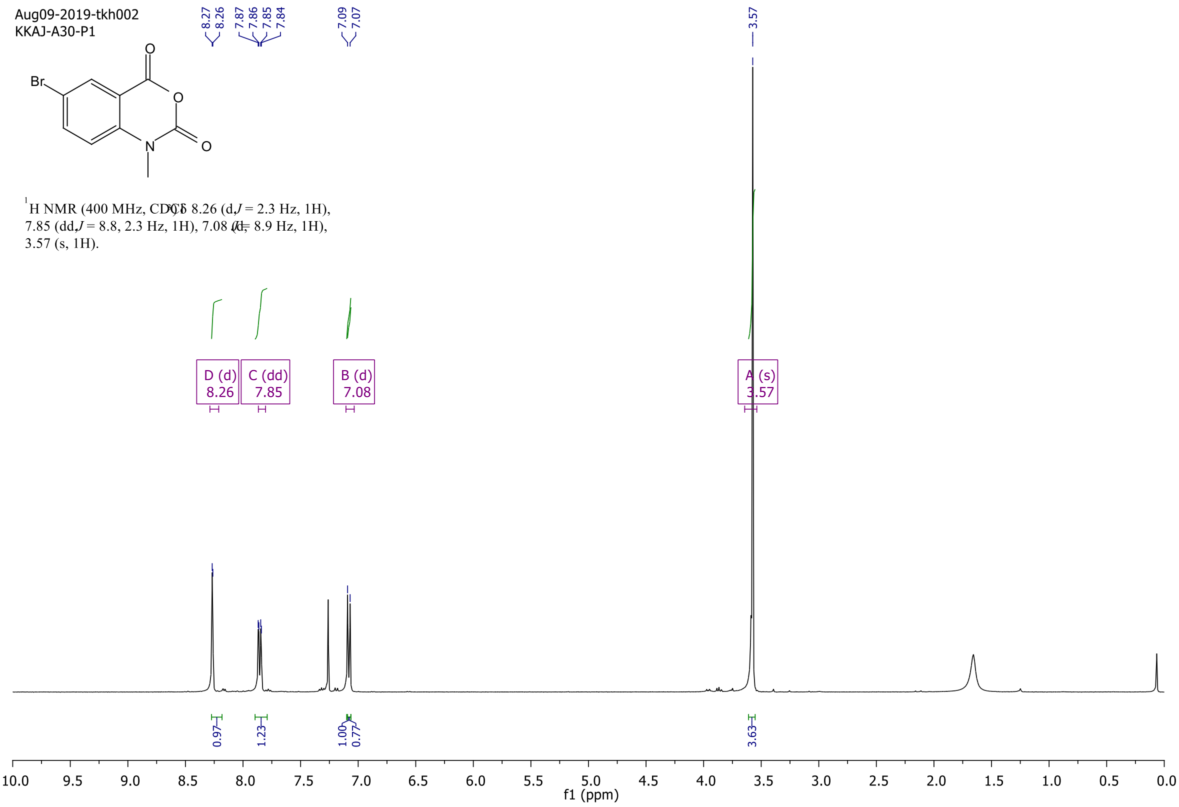

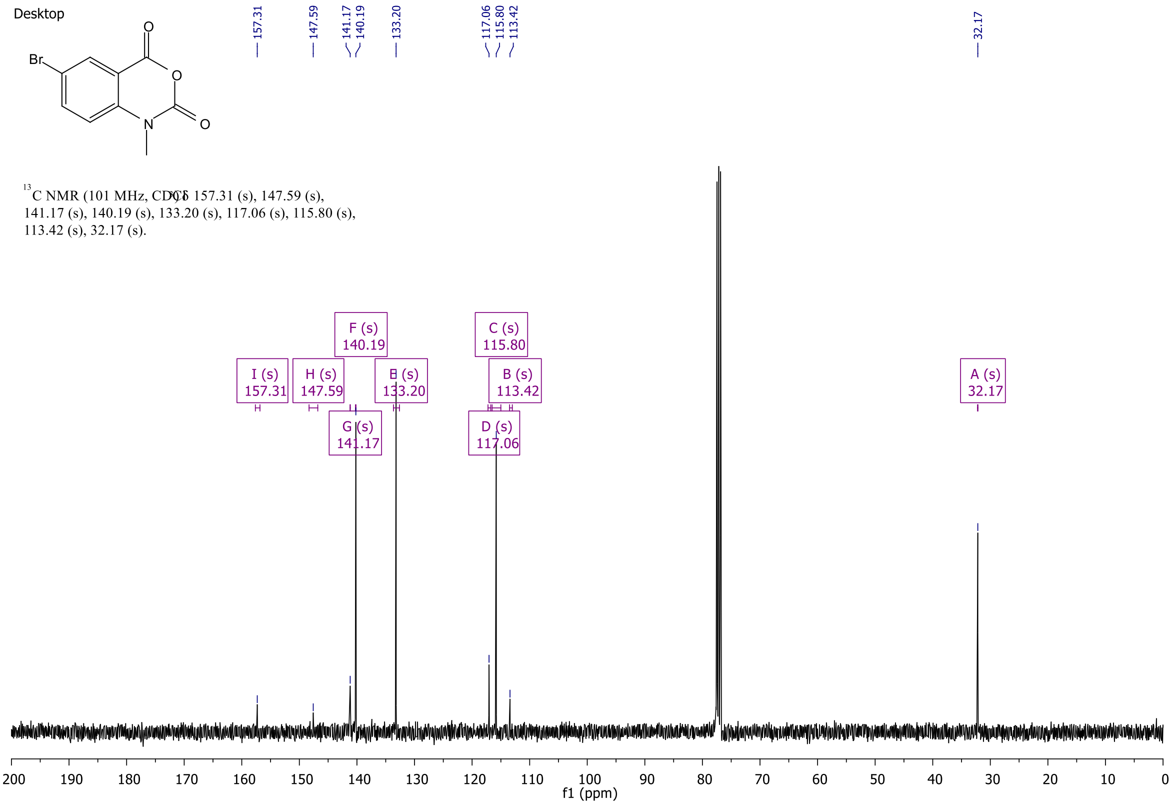

1-Ethyl-2H-benzo[d] [1,3] oxazine-2,4(1H)-dione (**1g**)

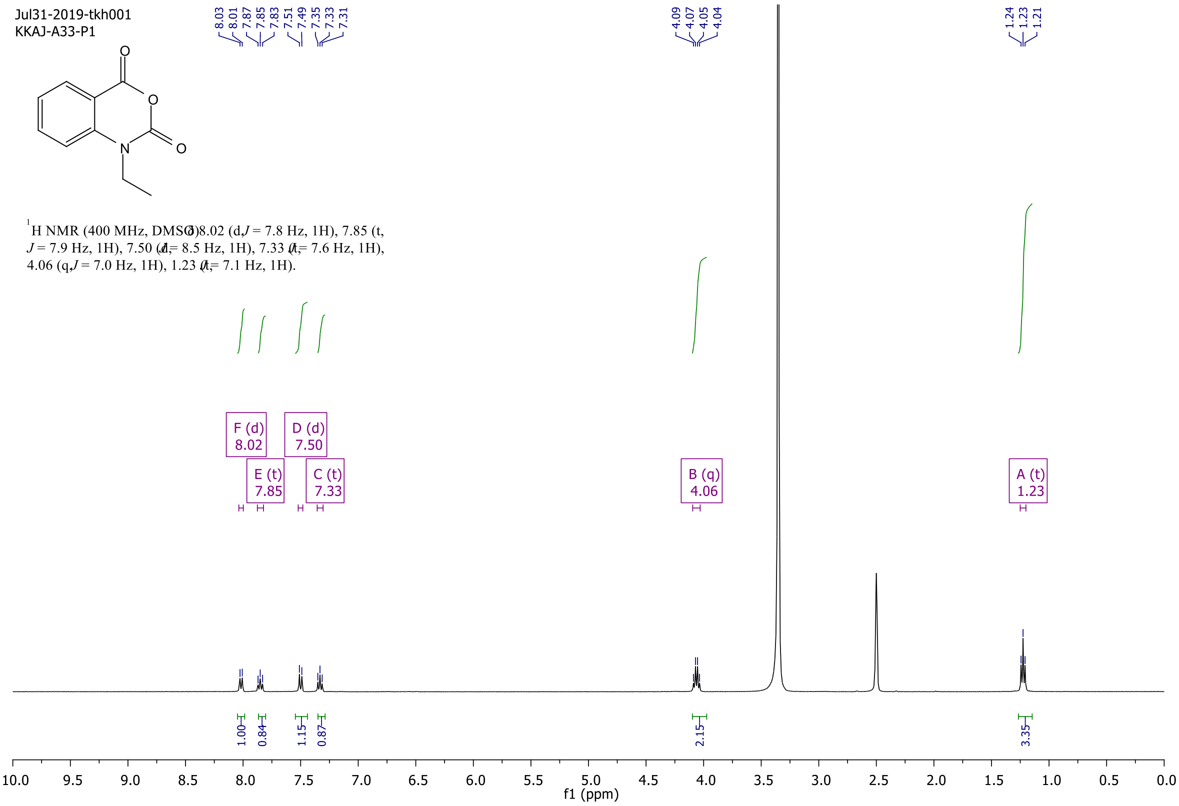

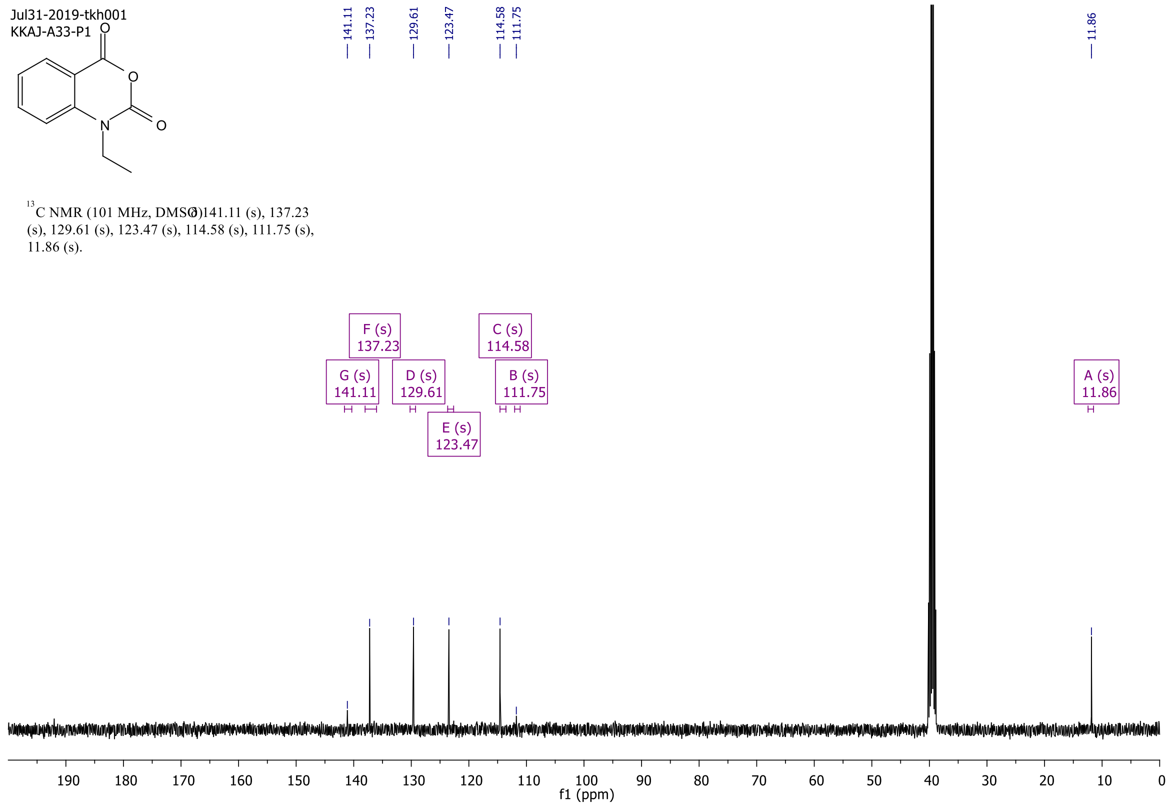

1.2 NMR spectra of 2a and 2b

Ethyl 3-oxohexadecanoate (**2a**)

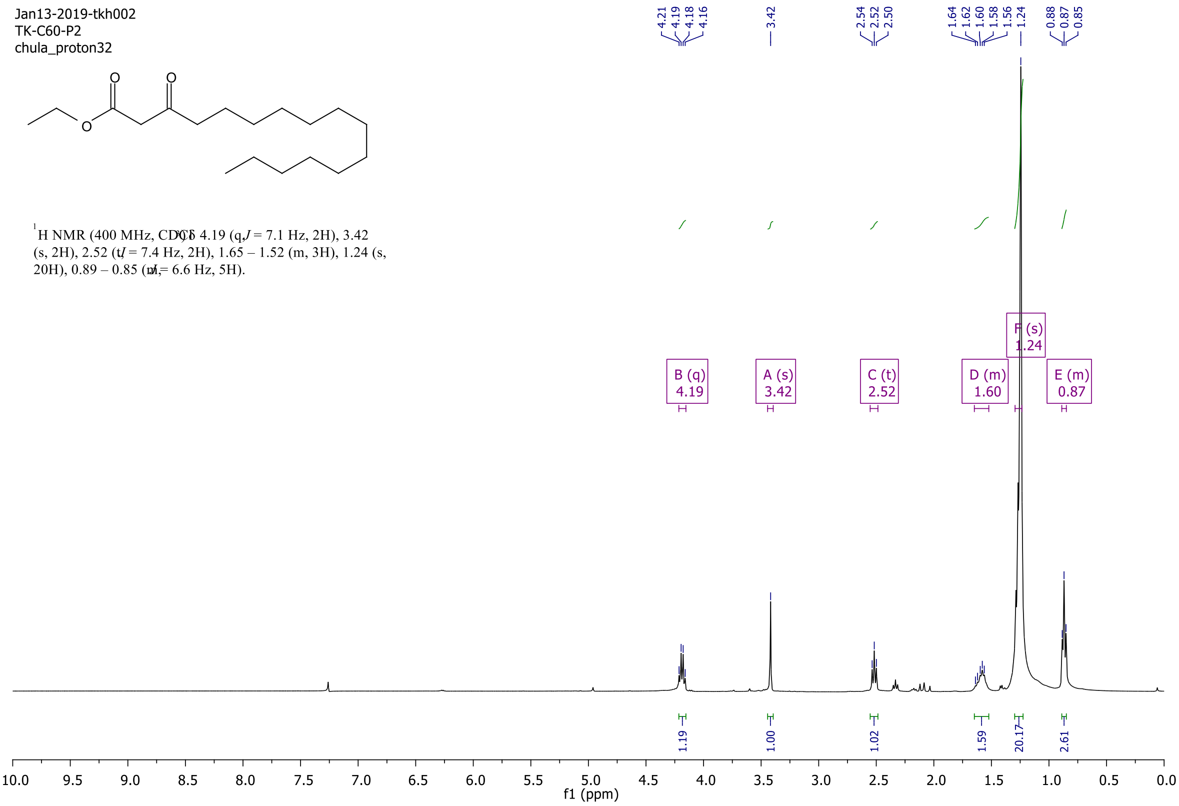

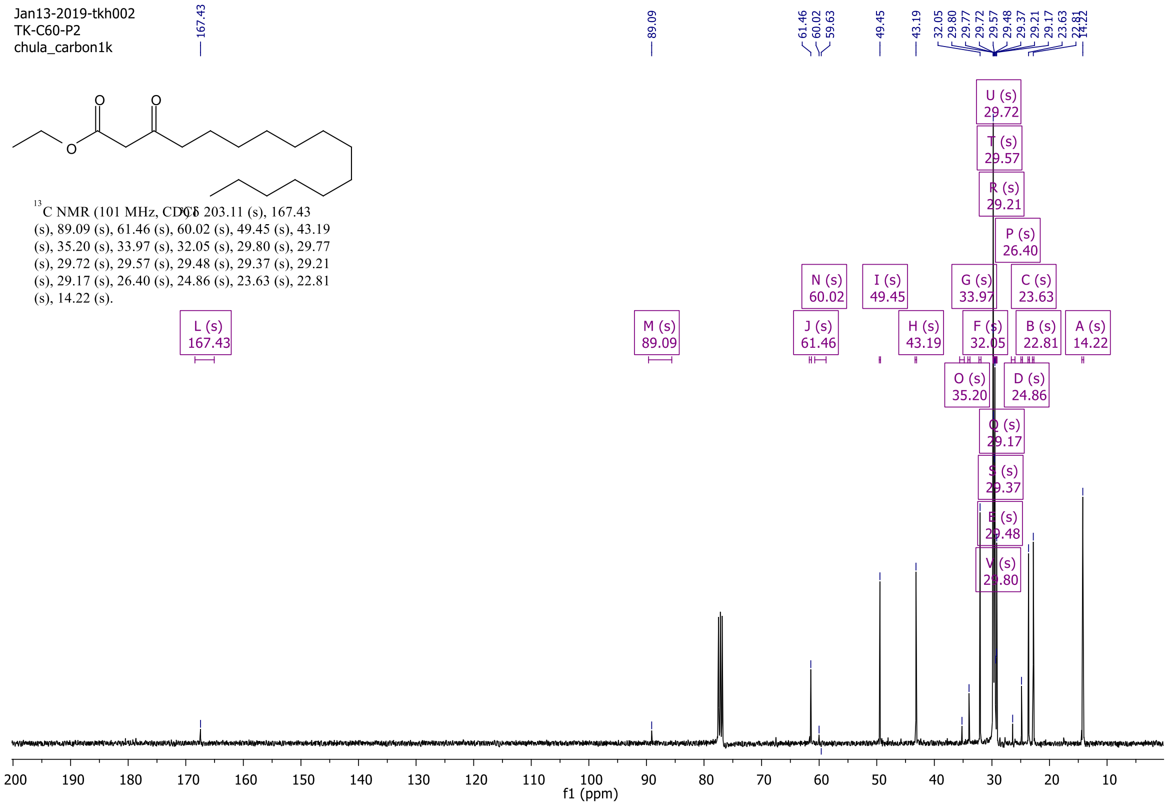

Ethyl 3-oxododecanoate (**2b**)

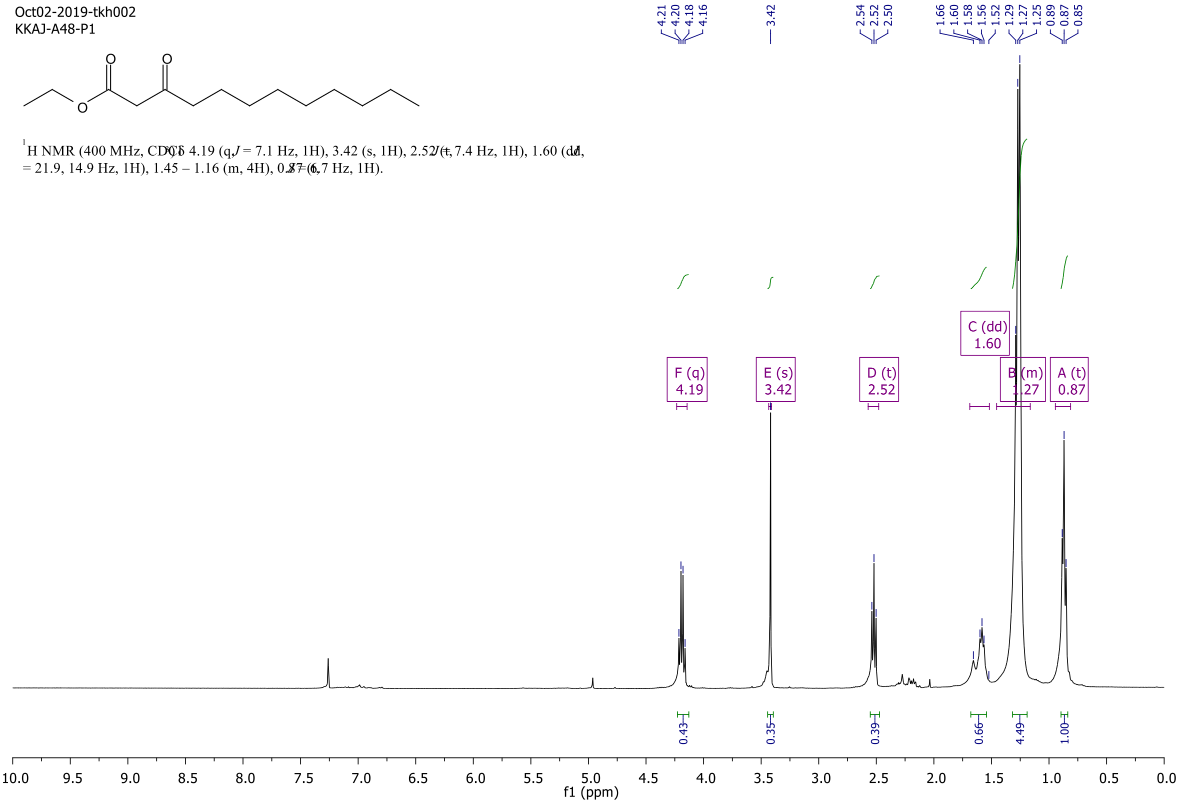

^{1}H NMR (400 MHz, $CDCl_3$) δ 4.19 (q, J = 7.1 Hz, 1H), 3.42 (s, 1H), 2.52 (t, J = 7.4 Hz, 1H), 1.60 (dd, J = 21.9, 14.9 Hz, 1H), 1.45 – 1.16 (m, 4H), 0.87 (t, J = 6.7 Hz, 1H).

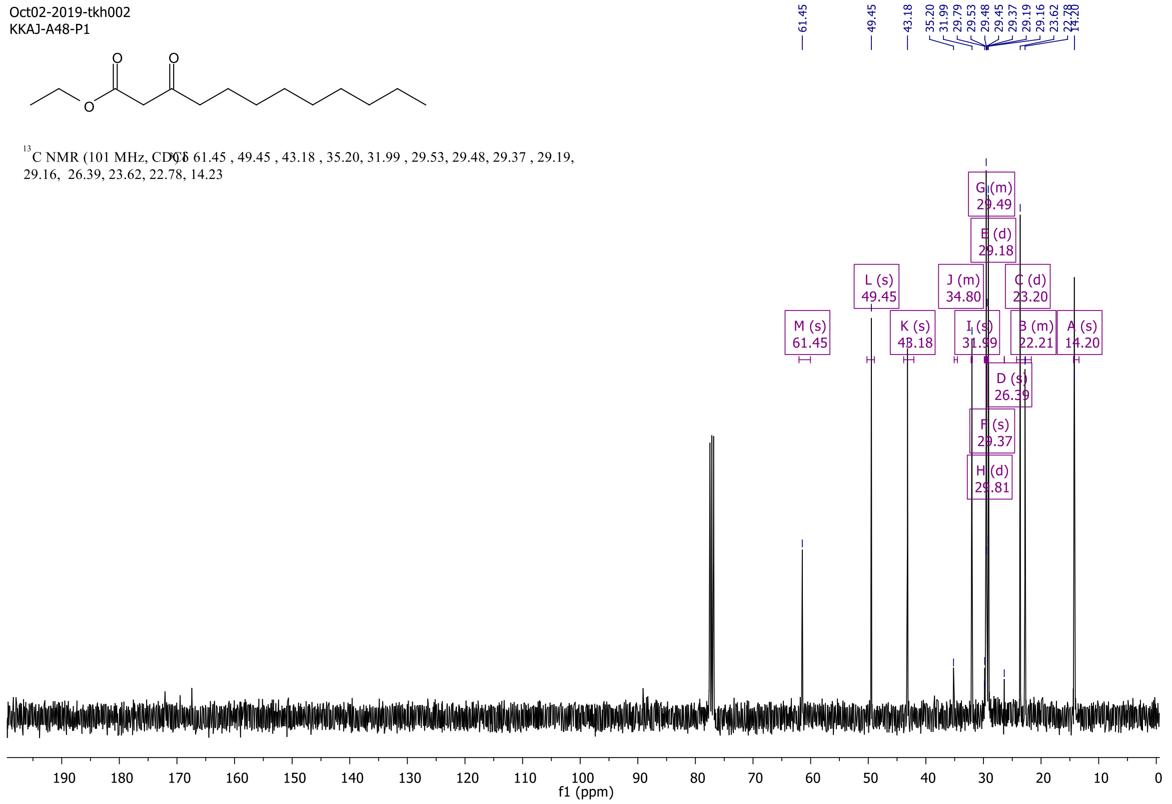

^{13}C NMR (101 MHz, $CDCl_3$) δ 61.45 , 49.45 , 43.18 , 35.20, 31.99 , 29.53, 29.48, 29.37 , 29.19, 29.16, 26.39, 23.62, 22.78, 14.23

1.3 NMR and HM spectra of 3a-3j, 4d and 5d

4-Hydroxy-1-methyl-3-tetradecanoylquinolin-2(1H)-one (**3a**)

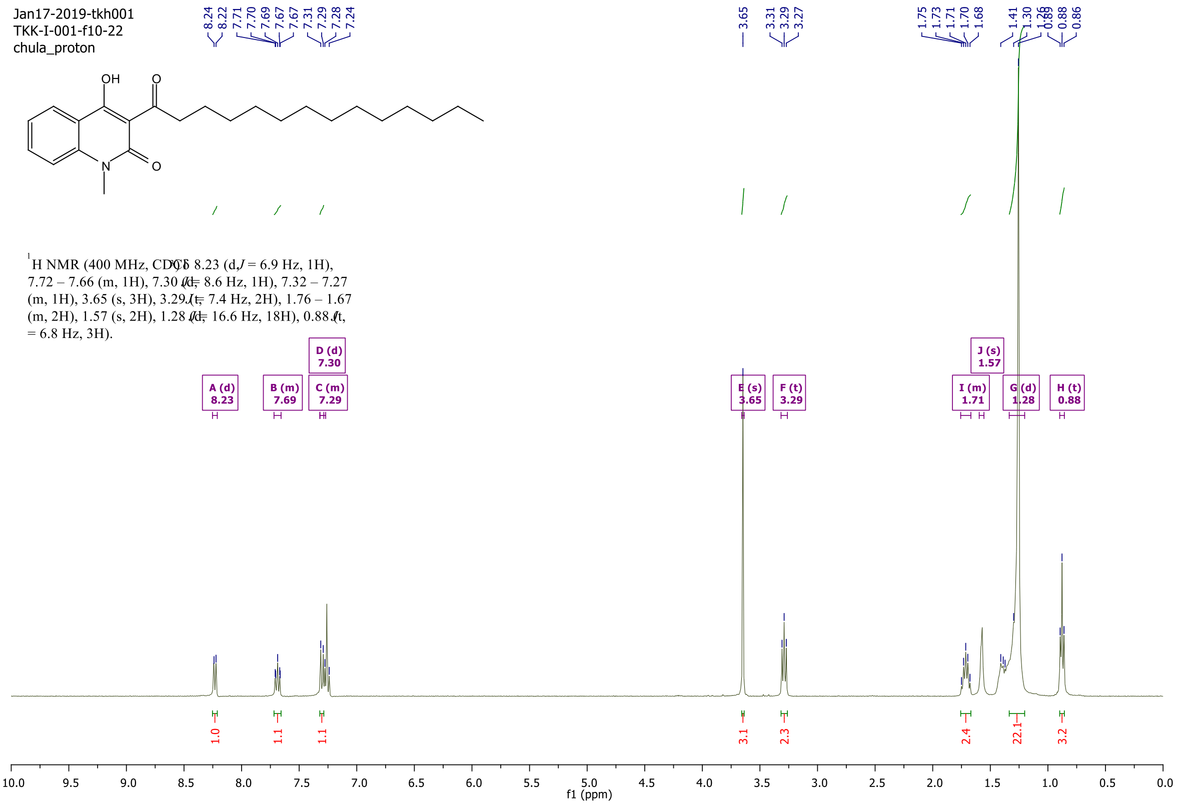

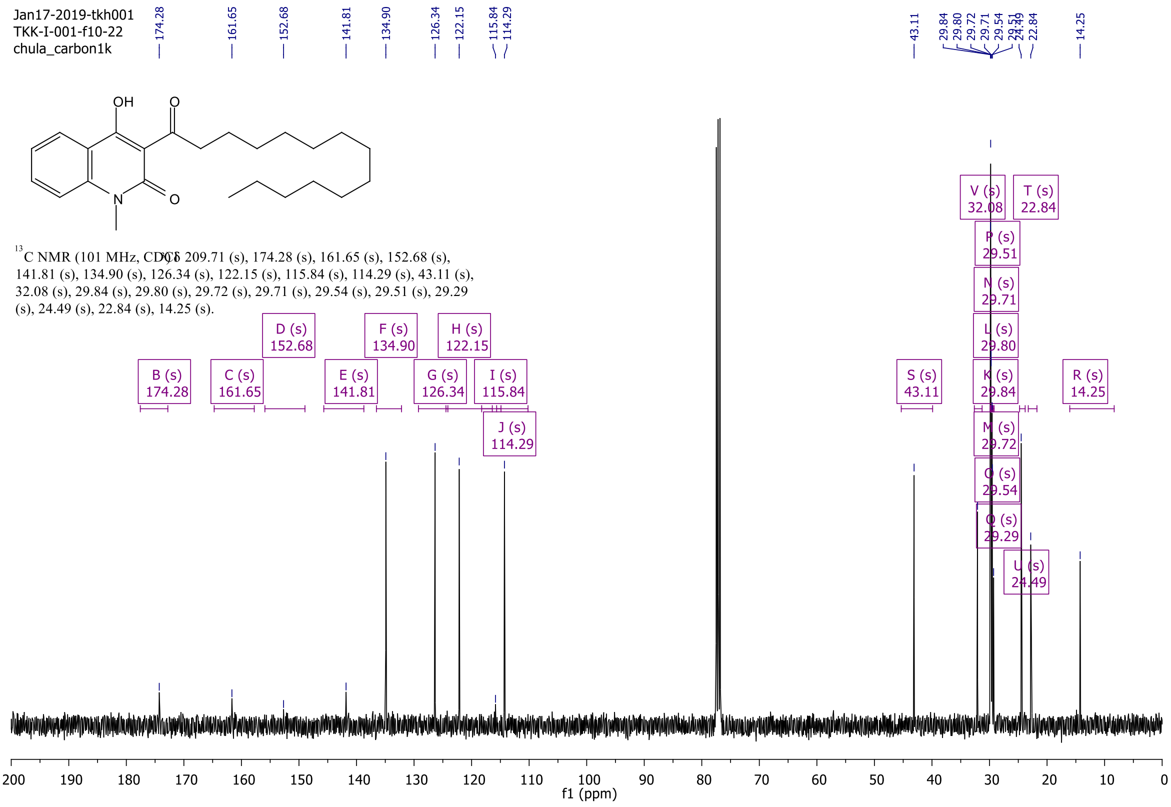

Generic Display Report

Analysis Info

Analysis Name	D:\Data\Data Service\190819\A16_RB6_01_2960.d	Acquisition Date	8/19/2019 7:31:14 PM
Method	nv_pos_6min_profile_wguardcol_190624.m	Operator	CU.
Sample Name	A16	Instrument	micrOTOF-Q II
Comment			

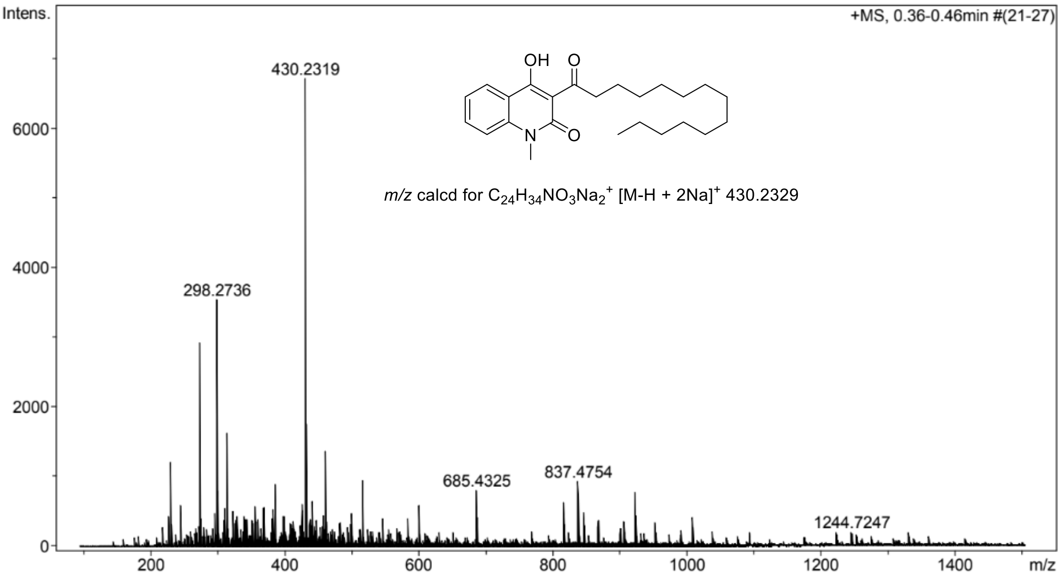

m/z calcd for $C_{24}H_{34}NO_3Na_2^+$ [M-H + 2Na]$^+$ 430.2329

4-Hydroxy-6-methoxy-1-methyl-3-tetradecanoylquinolin-2(1H)-one (**3b**)

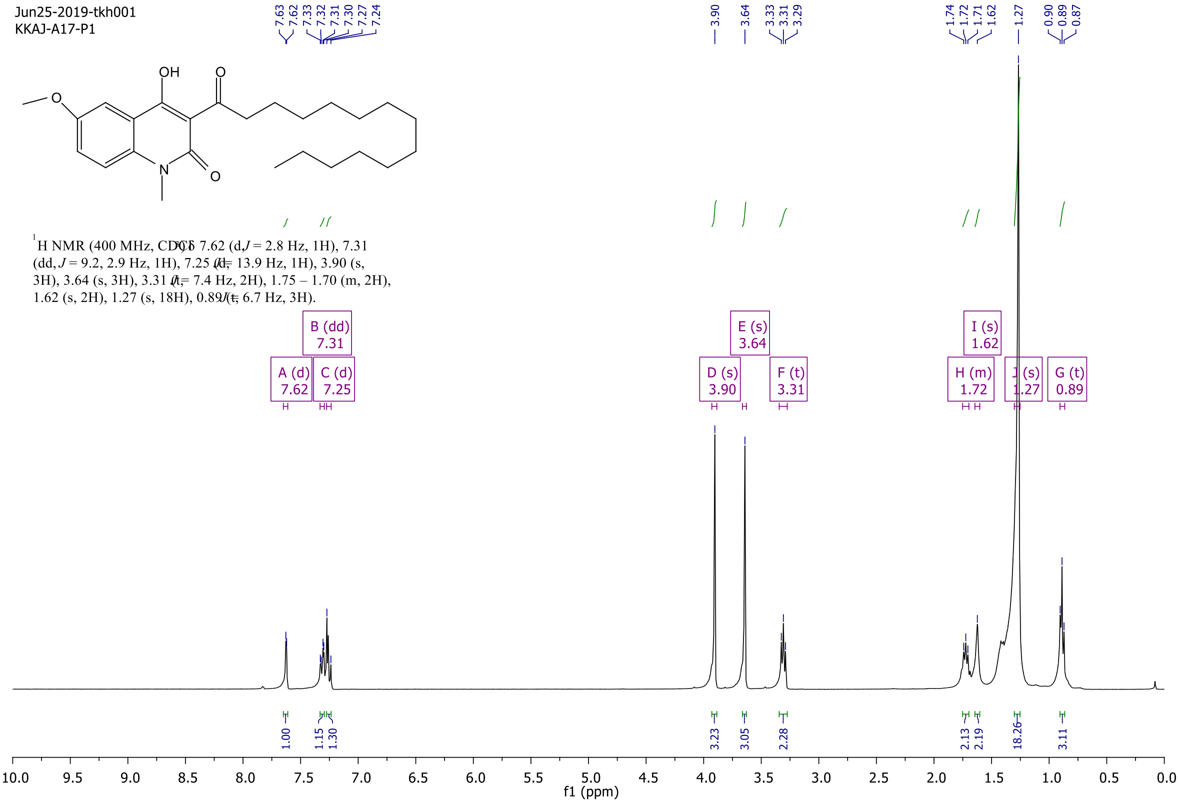

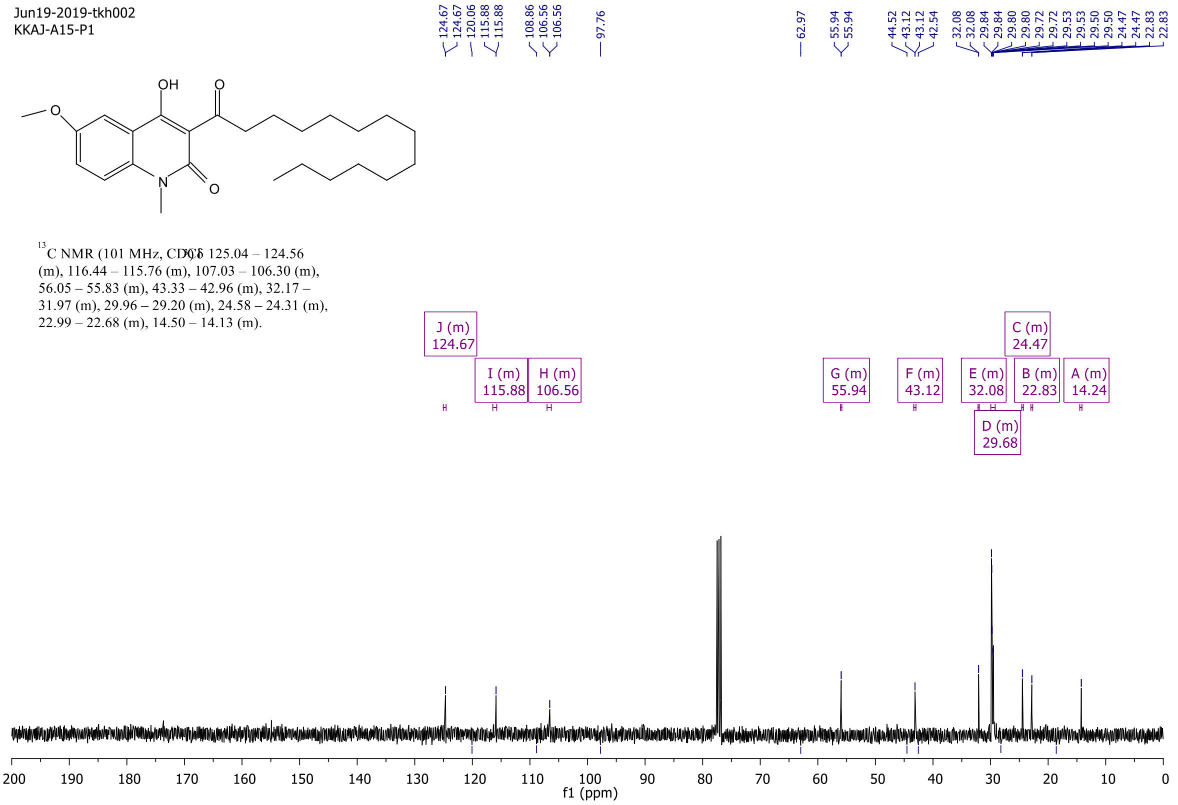

Generic Display Report

Analysis Info

Analysis Name	D:\Data\Data Service\190819\A17_RB5_01_2959.d	Acquisition Date	8/19/2019 7:24:49 PM
Method	nv_pos_6min_profile_wguardcol_190624.m	Operator	CU.
Sample Name	A17	Instrument	micrOTOF-Q II
Comment			

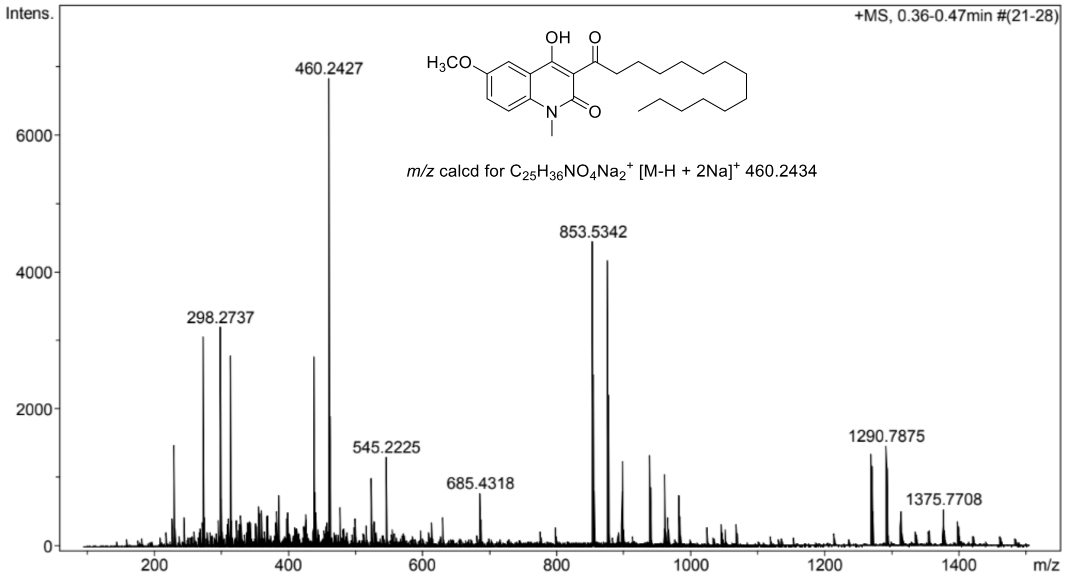

7-Chloro-4-hydroxy-1-methyl-3-tetradecanoylquinolin-2(1H)-one (**3c**)

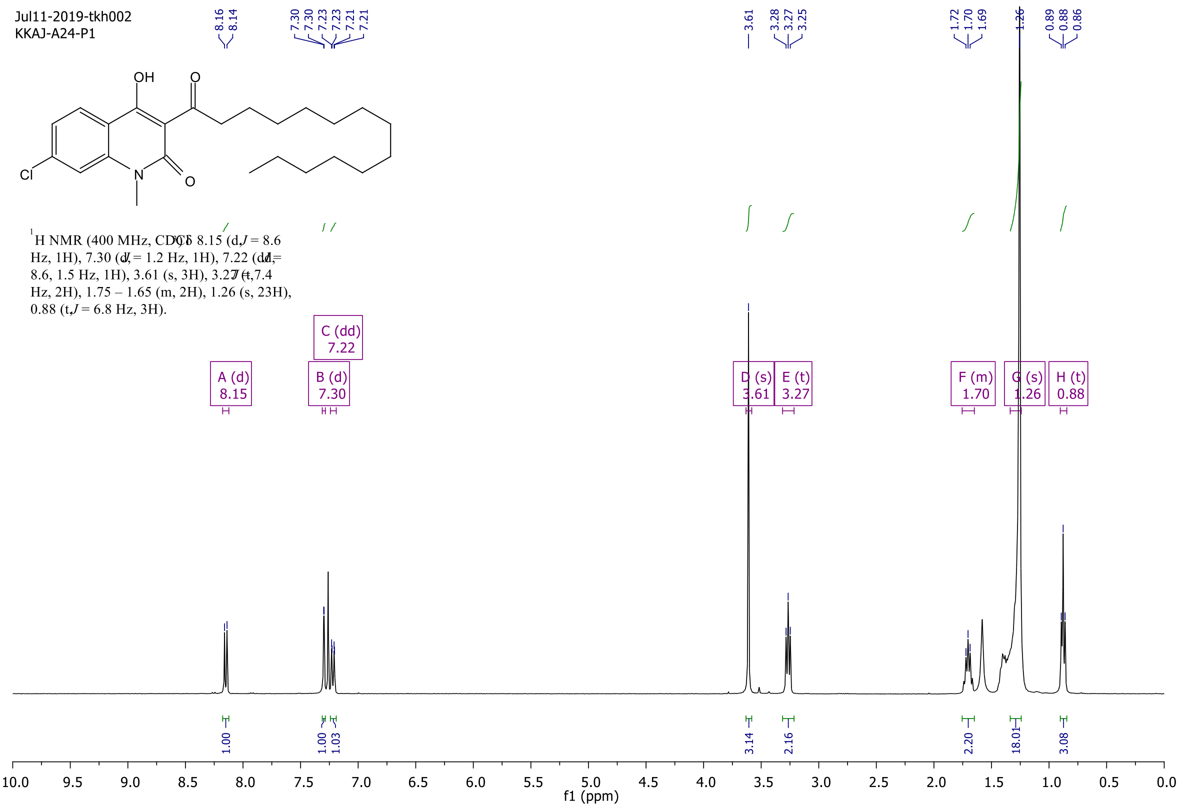

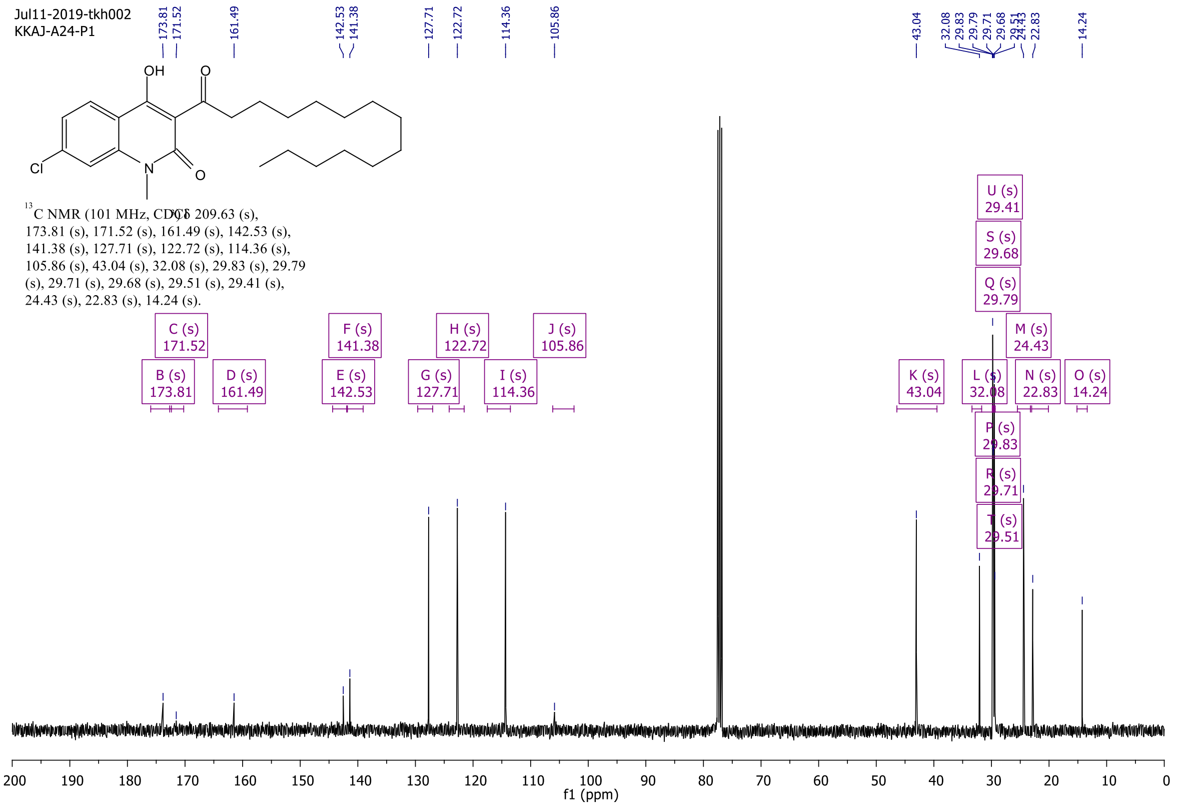

Generic Display Report

Analysis Info

Analysis Name	D:\Data\Data Service\190819\A24_RB7_01_2965.d	Acquisition Date	8/19/2019 8:05:53 PM
Method	nv_neg_6min_profile_wguardcol_190624.m	Operator	CU.
Sample Name	A24	Instrument	micrOTOF-Q II
Comment			

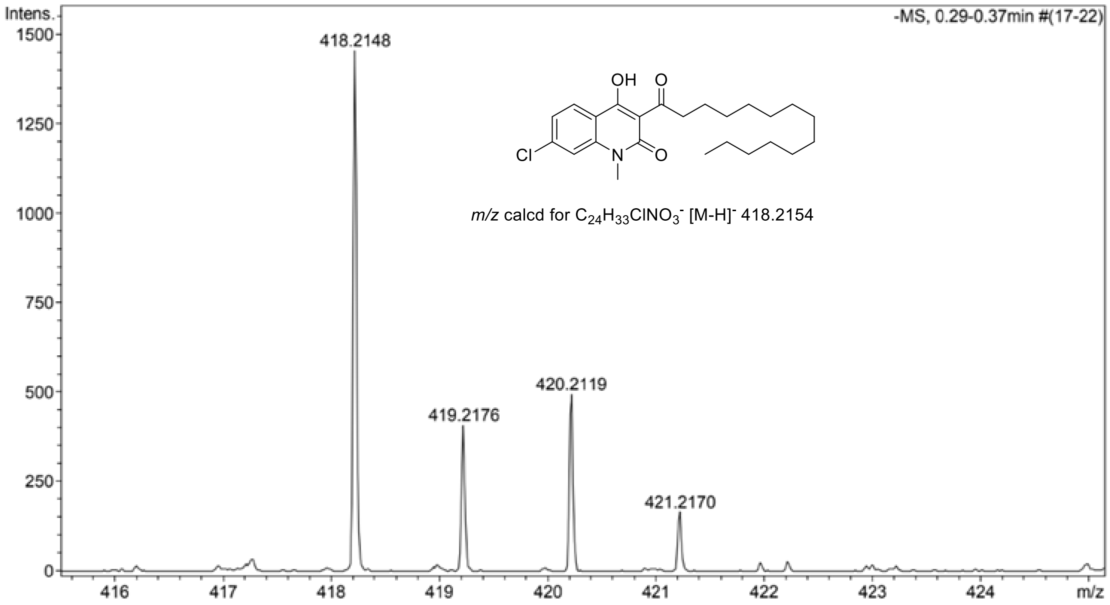

4-Hydroxy-1-methyl-7-nitro-3-tetradecanoylquinolin-2(1H)-one (**3d**)

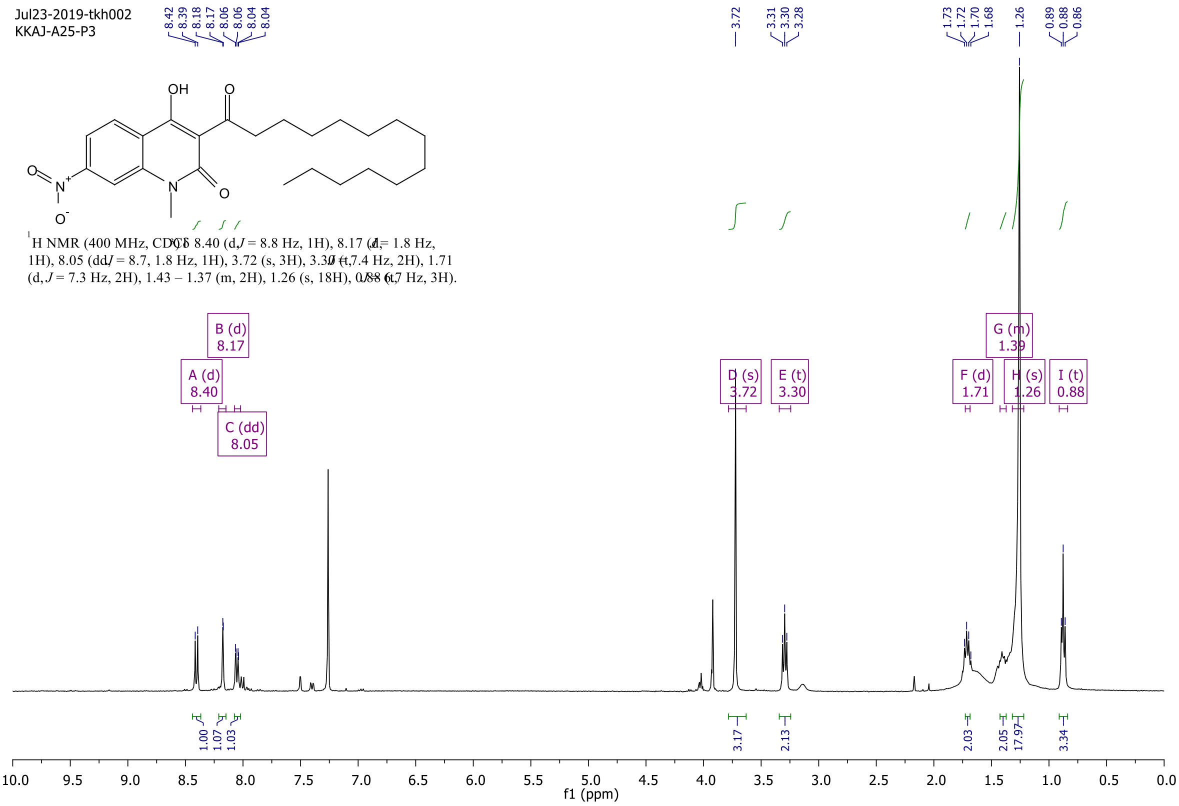

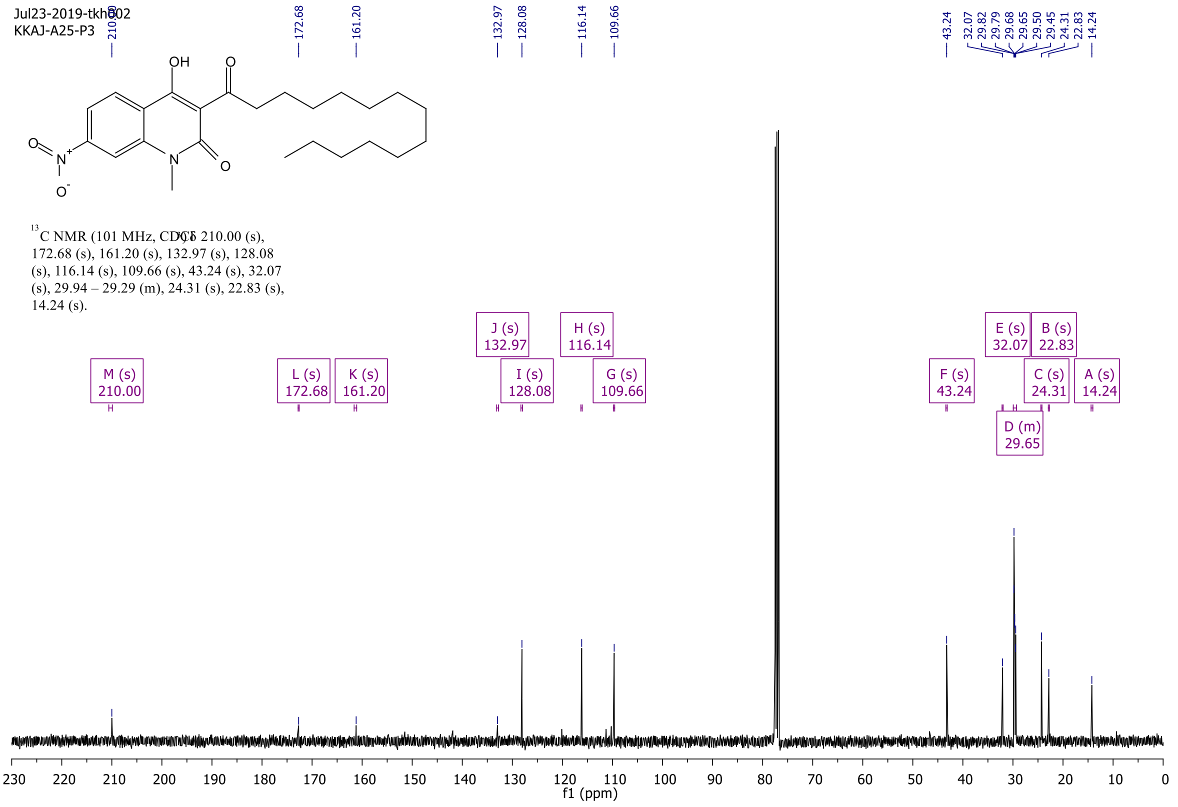

Generic Display Report

Analysis Info

Analysis Name	D:\Data\Data Service\190827\A25-P3_RA8_01_3015.d	Acquisition Date	8/27/2019 9:10:35 PM
Method	nv_neg_6min_profile_wguardcol_190624.m	Operator	CU.
Sample Name	A25-P3	Instrument	micrOTOF-Q II
Comment			

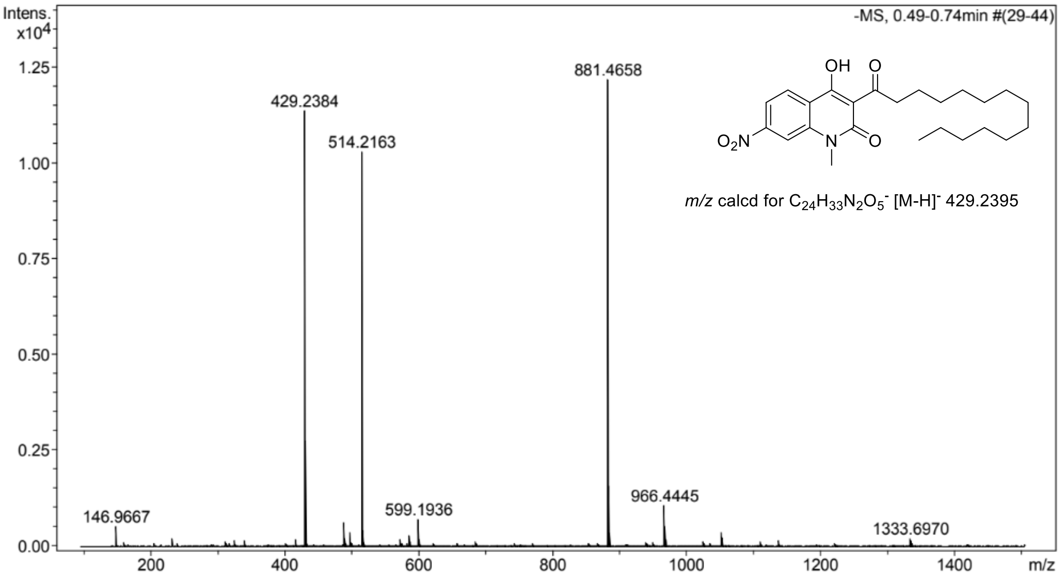

m/z calcd for $C_{24}H_{33}N_2O_5^-$ $[M\text{-}H]^-$ 429.2395

Ethyl 1-methyl-7-nitro-4-oxo-2-tridecyl-1,4-dihydroquinoline-3-carboxylate (**4d**)

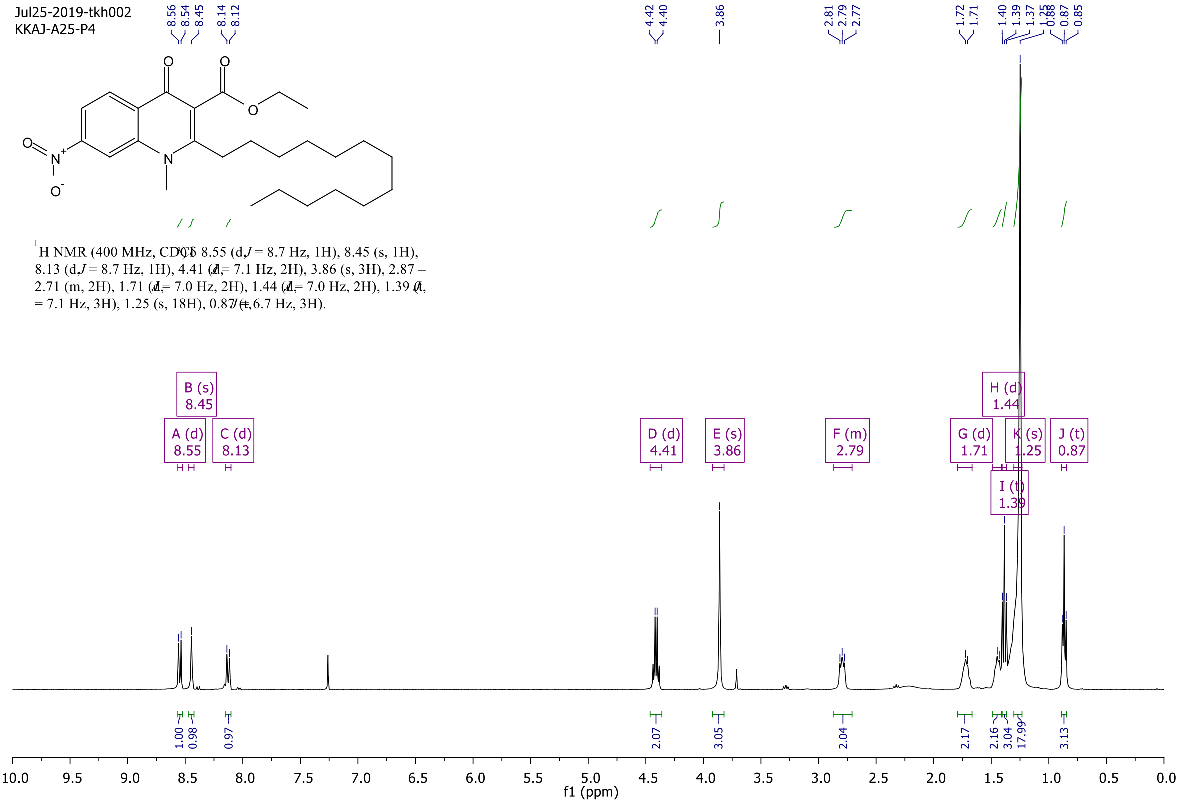

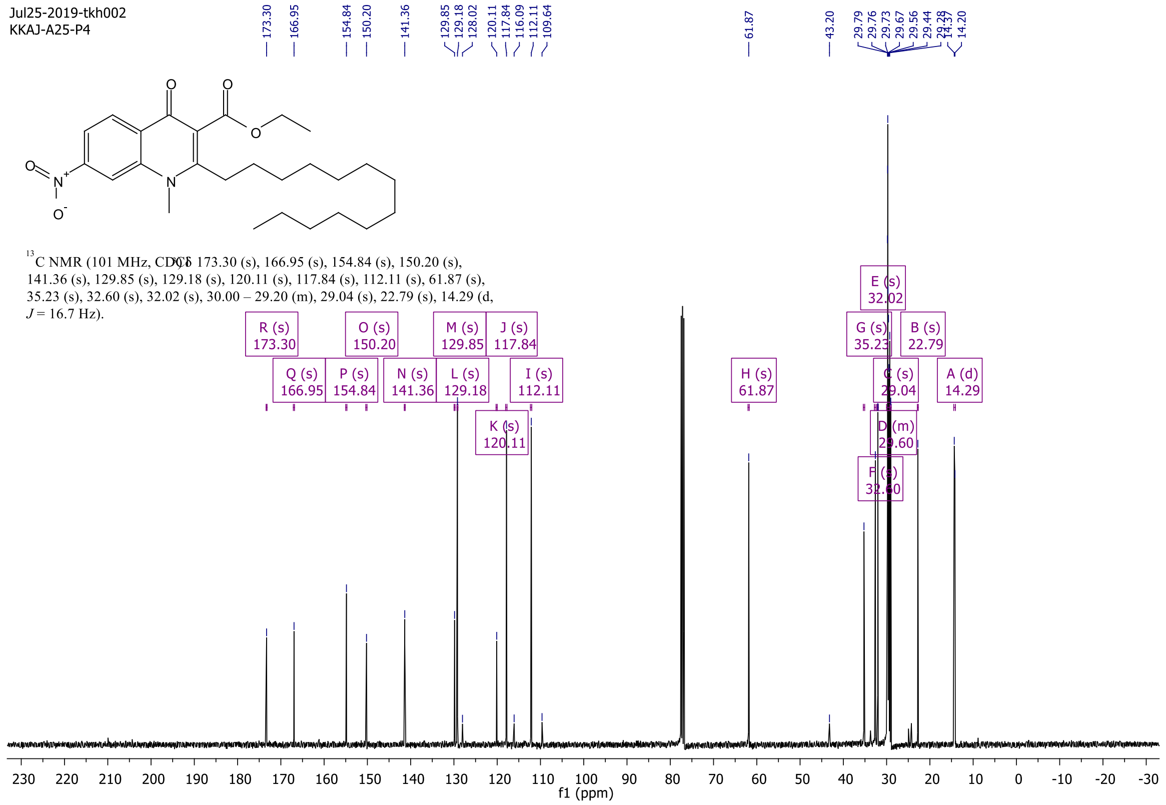

Generic Display Report

Analysis Info

Analysis Name	D:\Data\Data Service\190819\A25-P4_RB4_01_2958.d	Acquisition Date	8/19/2019 7:18:29 PM
Method	nv_pos_6min_profile_wguardcol_190624.m	Operator	CU.
Sample Name	A25-P4	Instrument	micrOTOF-Q II
Comment			

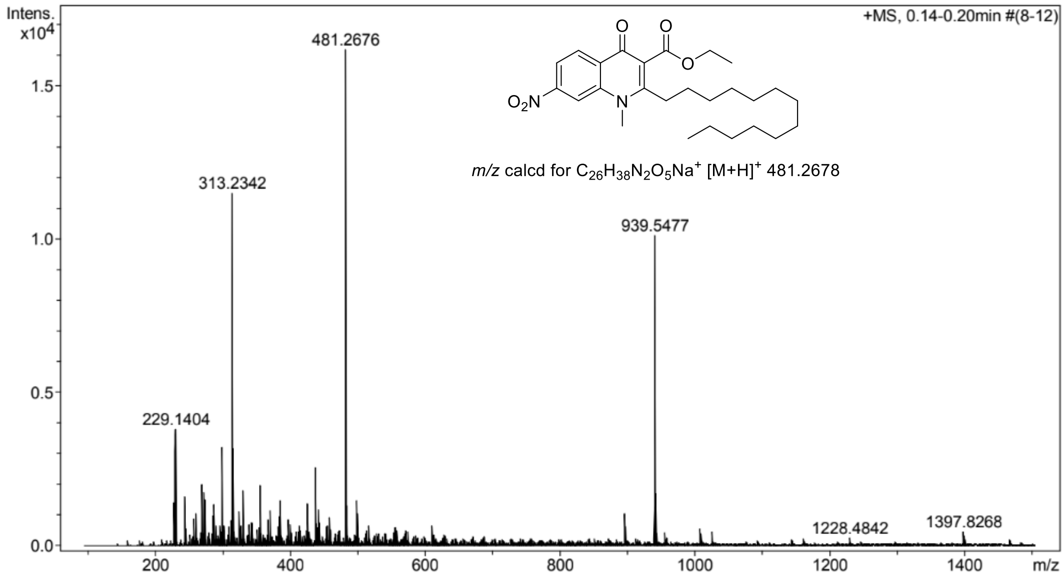

2-(methylamino)-4-nitrobenzoic acid (**5d**)

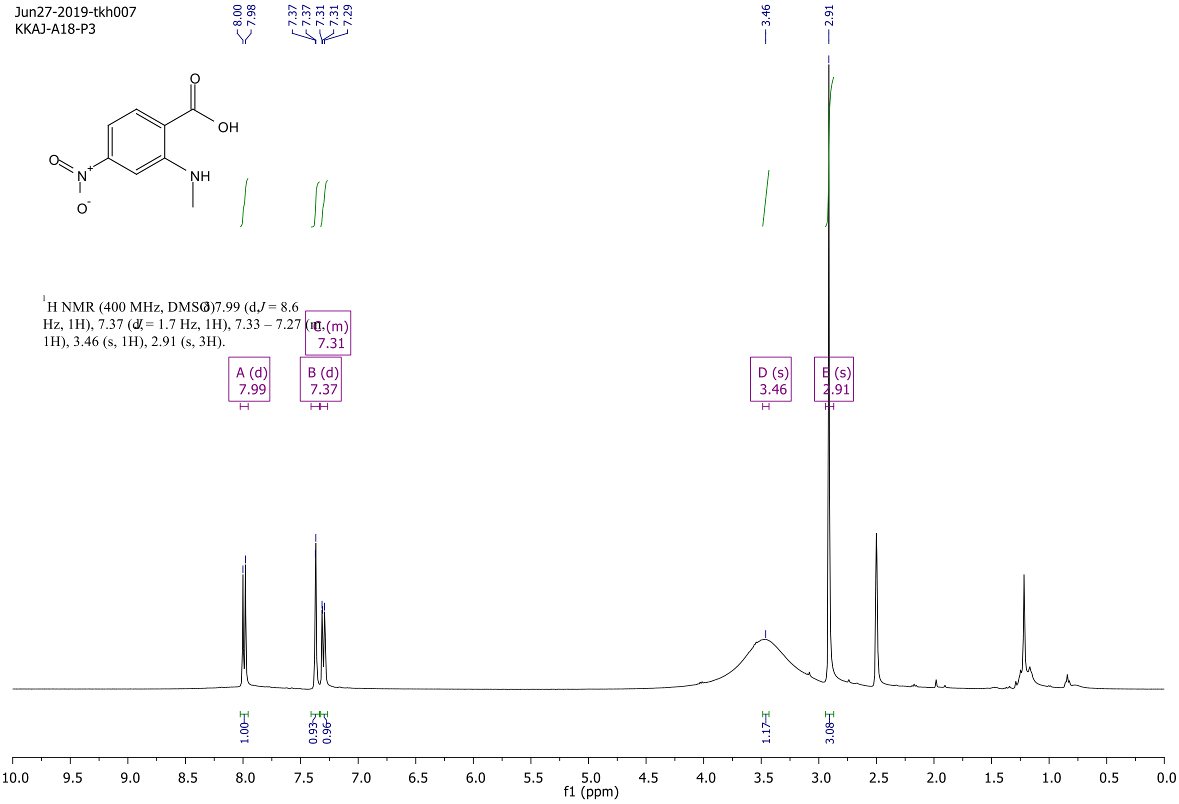

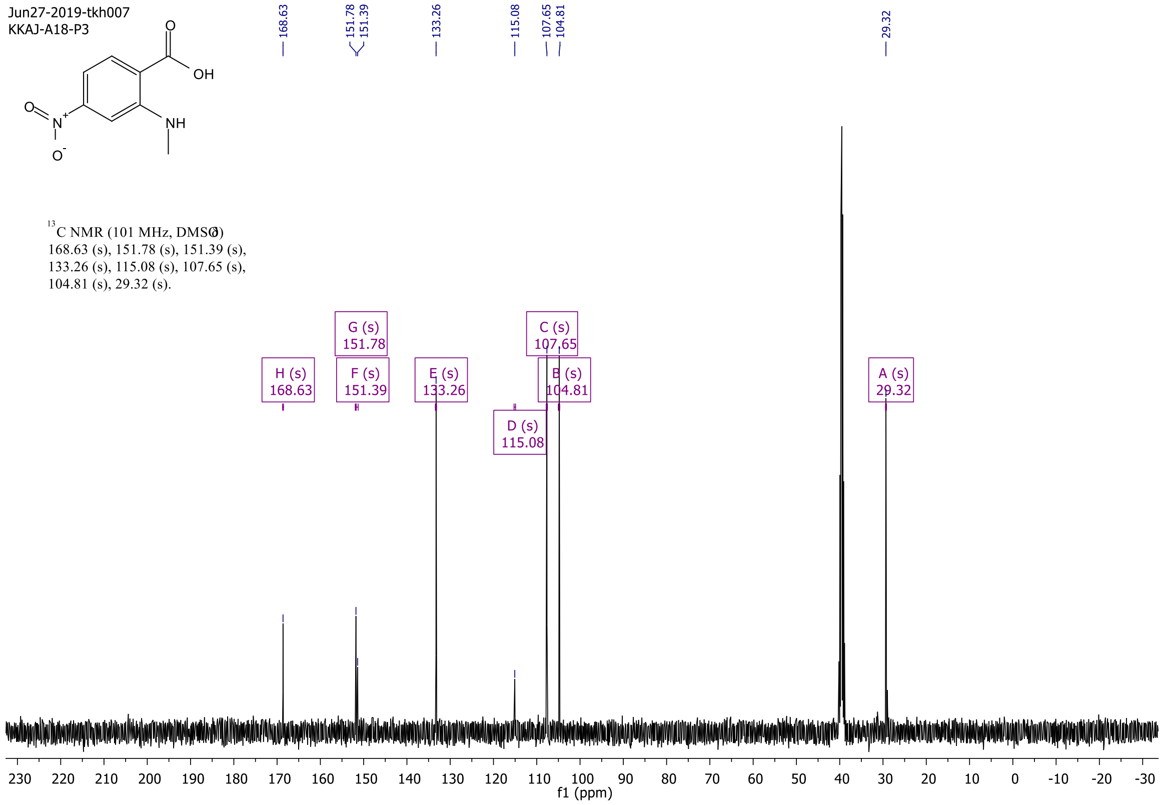

6-Chloro-4-hydroxy-1-methyl-3-tetradecanoylquinolin-2(1H)-one (**3e**)

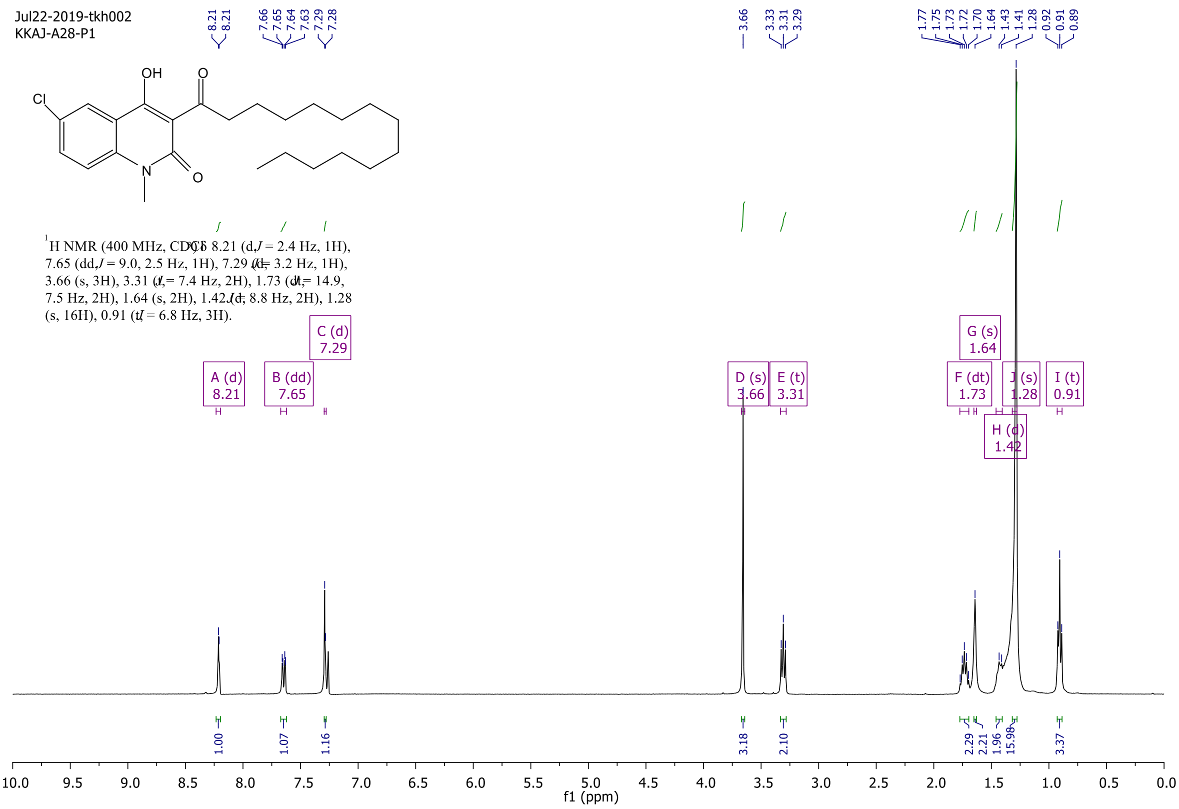

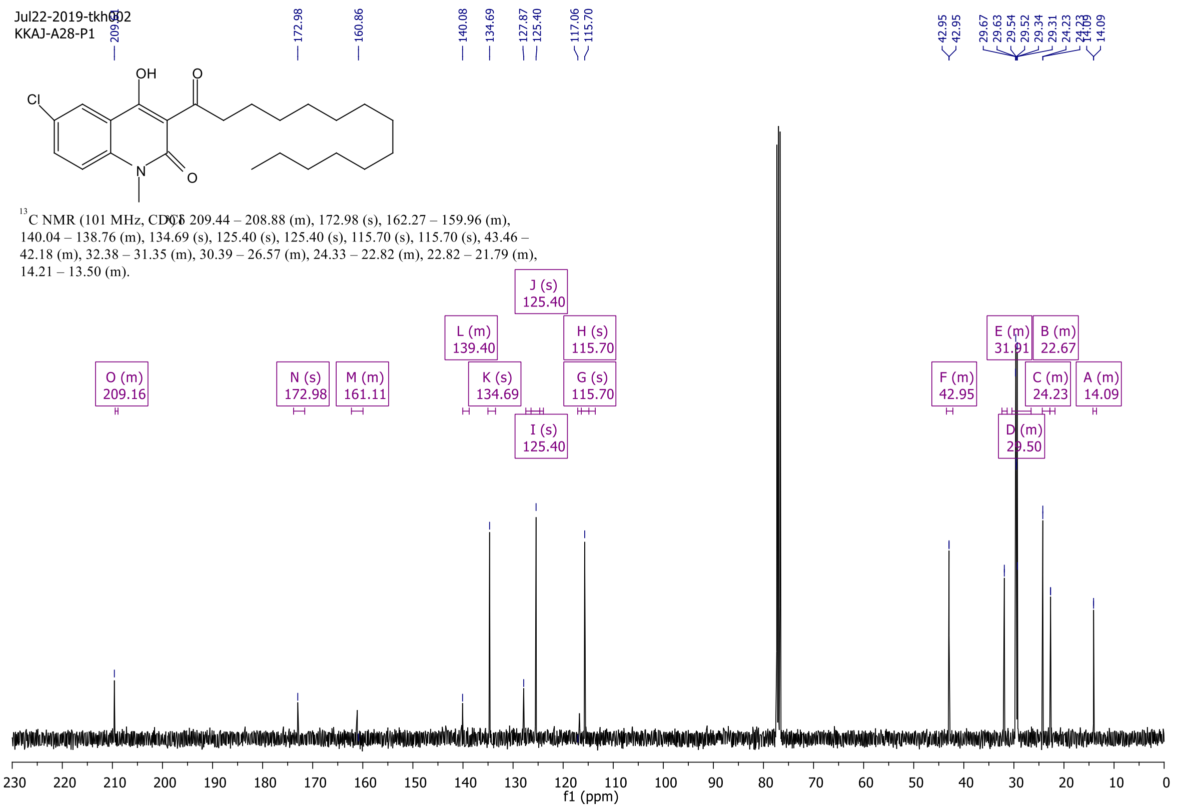

Generic Display Report

Analysis Info

Analysis Name D:\Data\Data Service\190819\A28_RB8_01_2967.d
Method nv_neg_6min_profile_wguardcol_190624.m
Sample Name A28
Comment

Acquisition Date 8/19/2019 8:18:49 PM

Operator CU.
Instrument micrOTOF-Q II

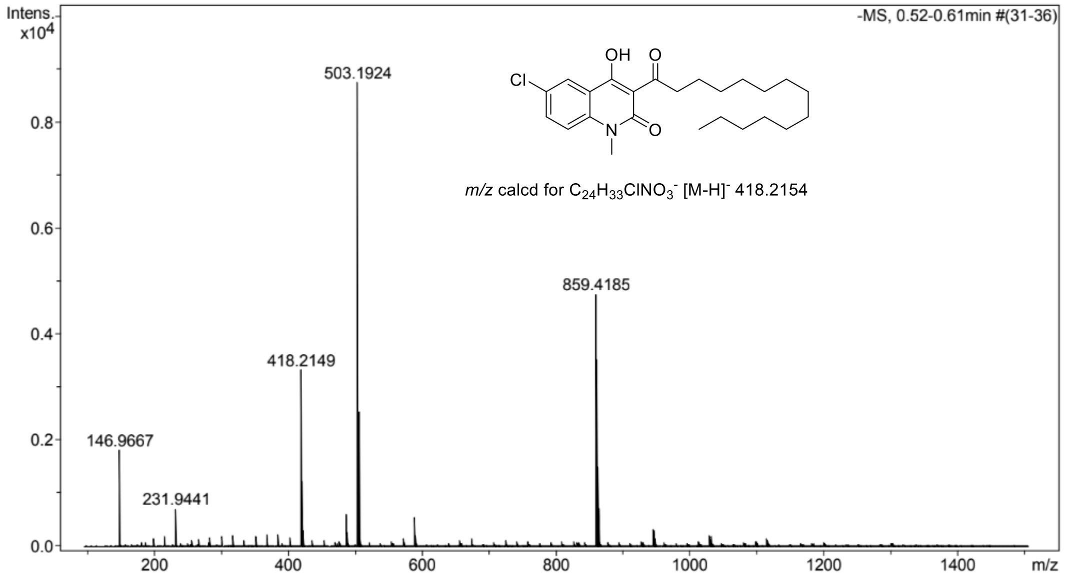

6-Bromo-4-hydroxy-1-methyl-3-tetradecanoylquinolin-2(1H)-one (**3f**)

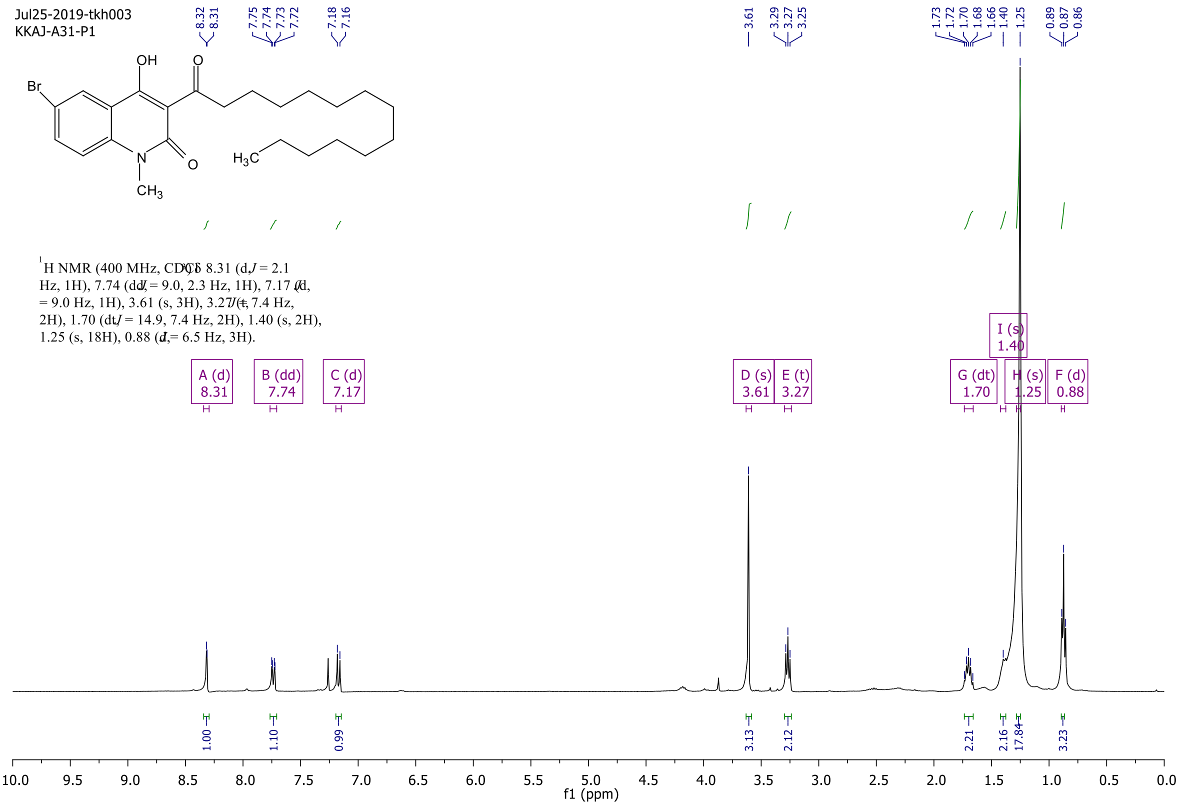

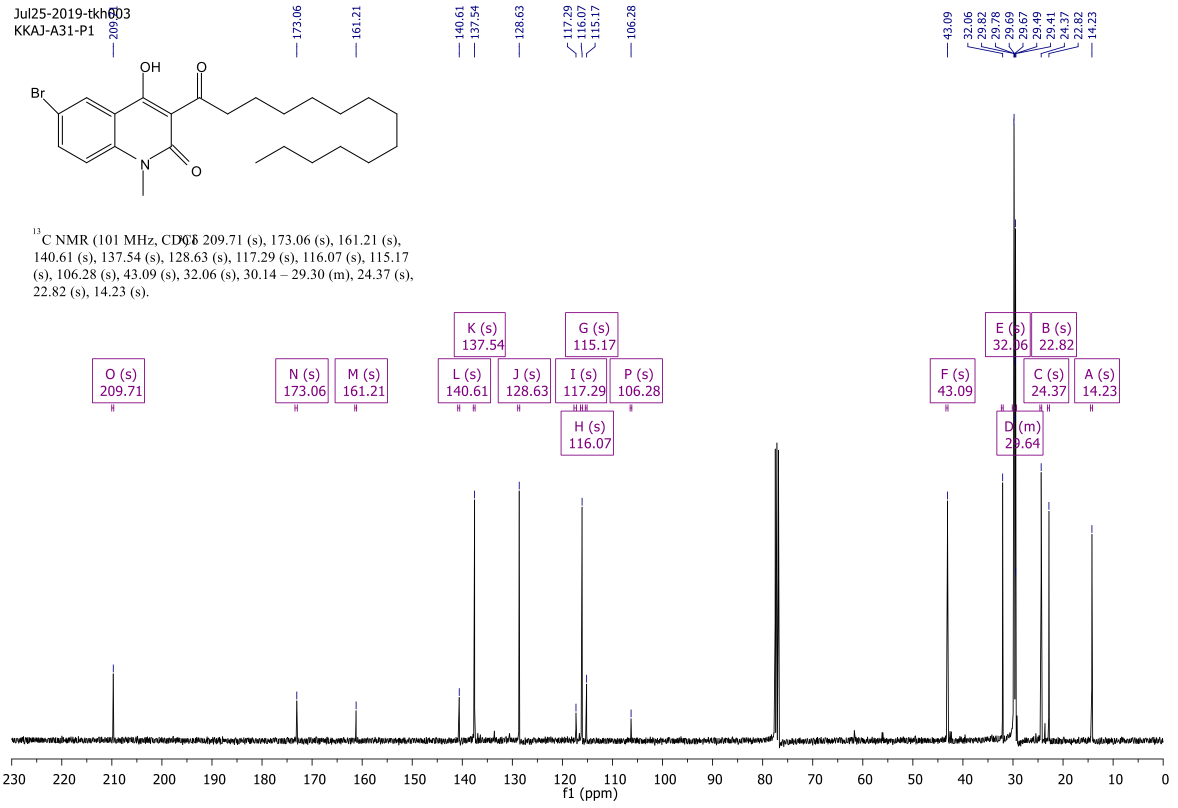

Generic Display Report

Analysis Info

Analysis Name	D:\Data\Data Service\190819\A31_RC2_01_2969.d	Acquisition Date	8/19/2019 8:31:48 PM
Method	nv_neg_6min_profile_wguardcol_190624.m	Operator	CU.
Sample Name	A31	Instrument	micrOTOF-Q II
Comment			

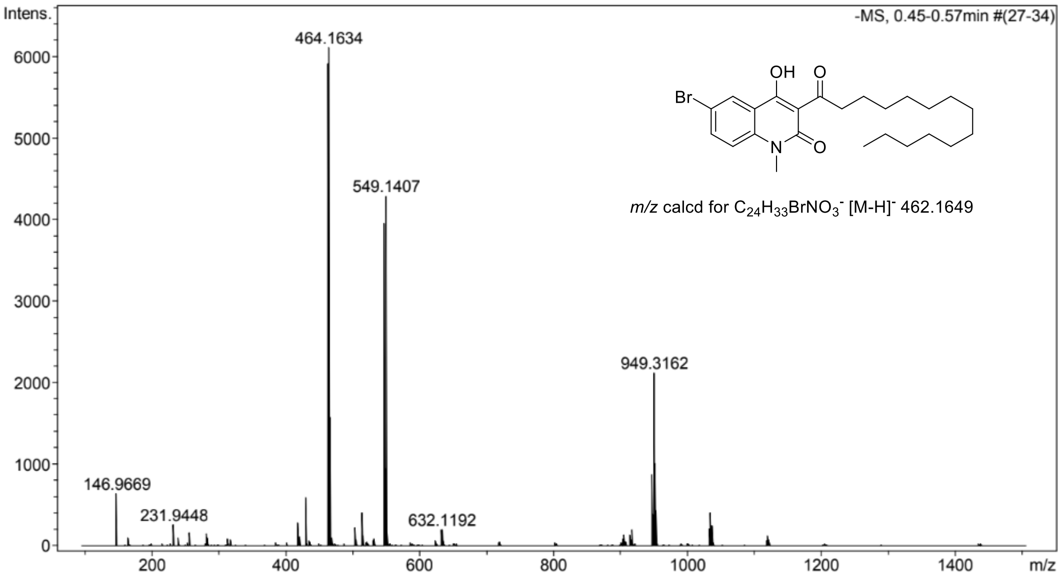

m/z calcd for $C_{24}H_{33}BrNO_3^-$ $[M-H]^-$ 462.1649

1-Ethyl-4-hydroxy-3-tetradecanoylquinolin-2(1H)-one (**3g**)

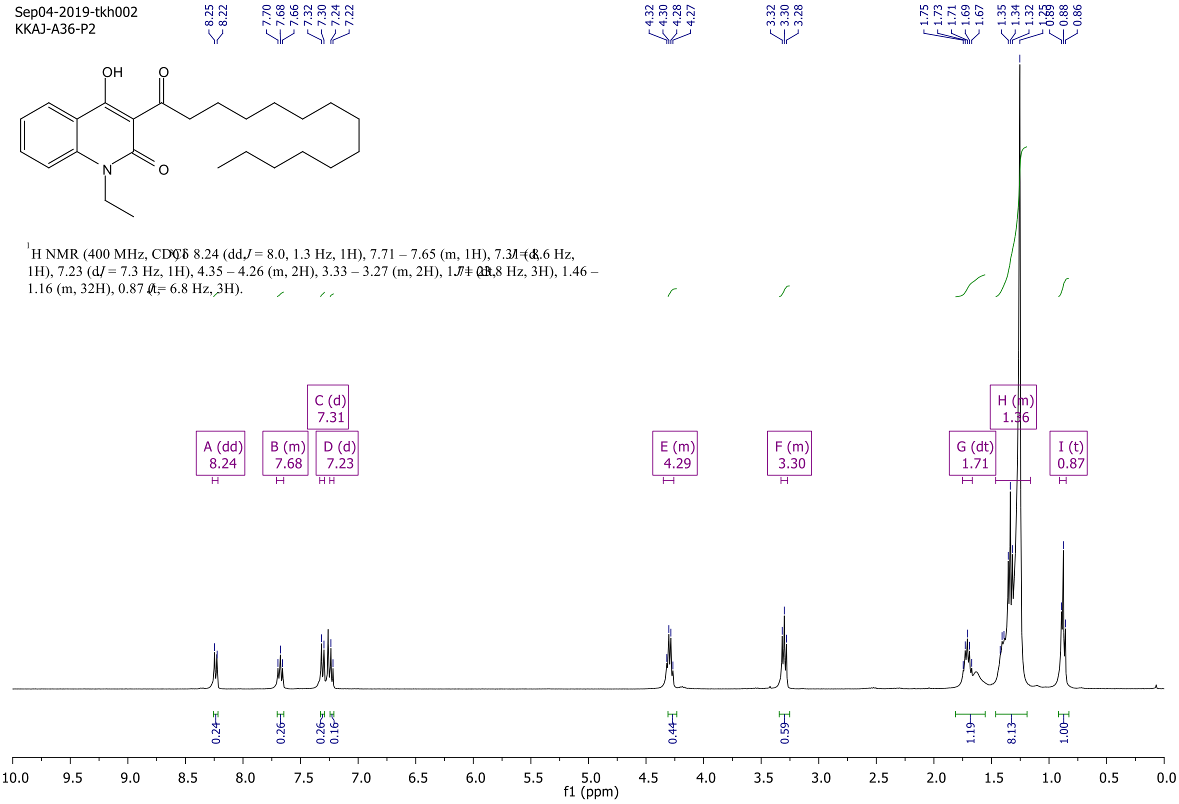

^{1}H NMR (400 MHz, $CDCl_3$) δ 8.24 (dd, J = 8.0, 1.3 Hz, 1H), 7.71 – 7.65 (m, 1H), 7.31 (d, J = 8.6 Hz, 1H), 7.23 (d, J = 7.3 Hz, 1H), 4.35 – 4.26 (m, 2H), 3.33 – 3.27 (m, 2H), 1.71 (dt, J = [illegible] Hz, 3H), 1.46 – 1.16 (m, 32H), 0.87 (t, J = 6.8 Hz, 3H).

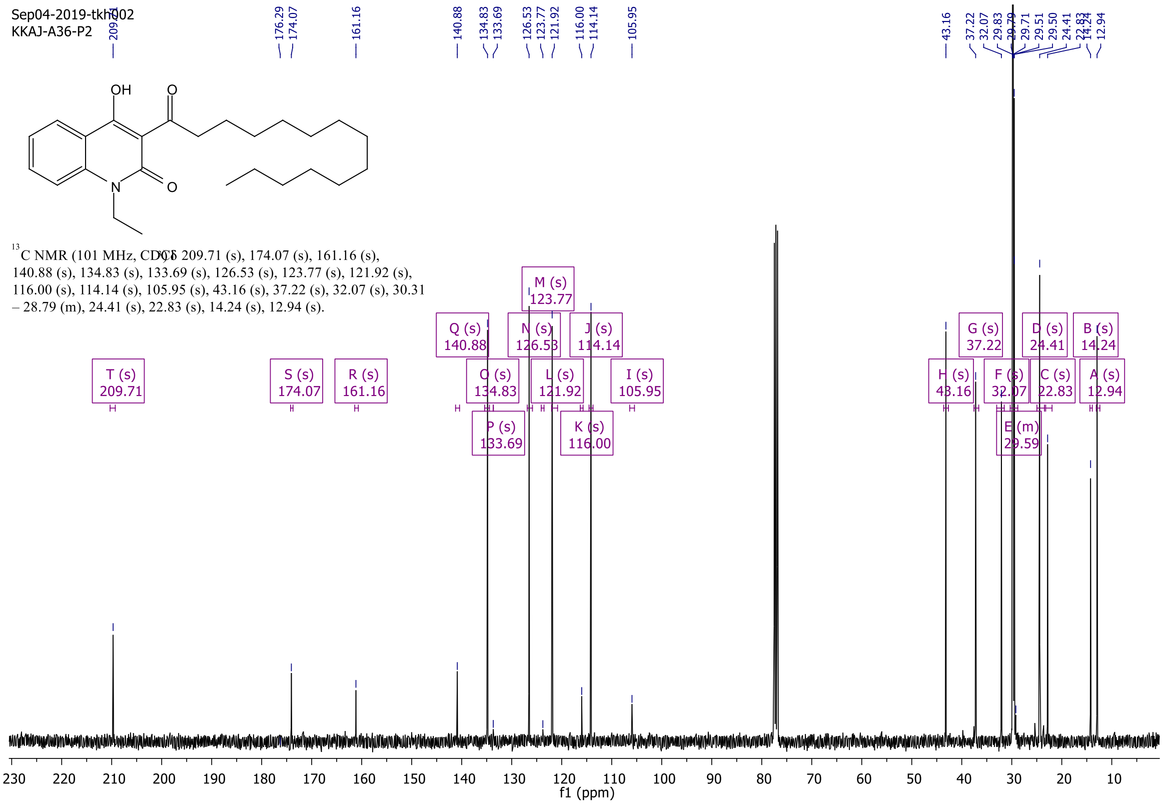

^{13}C NMR (101 MHz, $CDCl_3$) δ 209.71 (s), 174.07 (s), 161.16 (s), 140.88 (s), 134.83 (s), 133.69 (s), 126.53 (s), 123.77 (s), 121.92 (s), 116.00 (s), 114.14 (s), 105.95 (s), 43.16 (s), 37.22 (s), 32.07 (s), 30.31 – 28.79 (m), 24.41 (s), 22.83 (s), 14.24 (s), 12.94 (s).

BIORESOURCES RESEARCH UNIT

High resolution report

Analysis Name	D:\Data\customer\TK3g.d	Acquisition Date	6/1/2020 10:30:54 AM
Method	NaFormate_pos.m	Operator	Sutichai Ext: 3560
Sample Name	TK3g	Instrument	micrOTOF Bruker
		Calibrate by	Sodium Formate

Acquisition Parameter

Source Type	ESI	Ion Polarity	Positive	Set Nebulizer	0.3 Bar
Focus	Not active			Set Dry Heater	180 ?C
Scan Begin	100 m/z	Set Capillary	4500 V	Set Dry Gas	4.0 l/min
Scan End	2000 m/z	Set End Plate Offset	-500 V	Set Divert Valve	Source

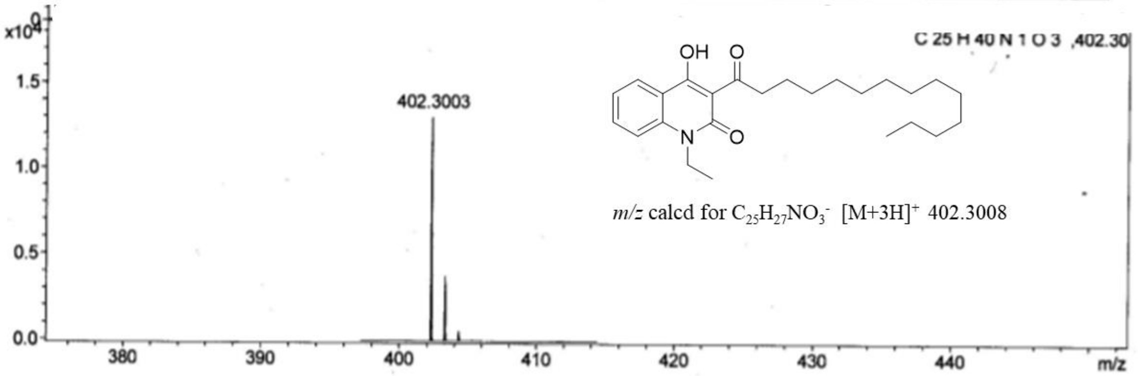

3-Decanoyl-4-hydroxy-1-methylquinolin-2(1H)-one (**3h**)

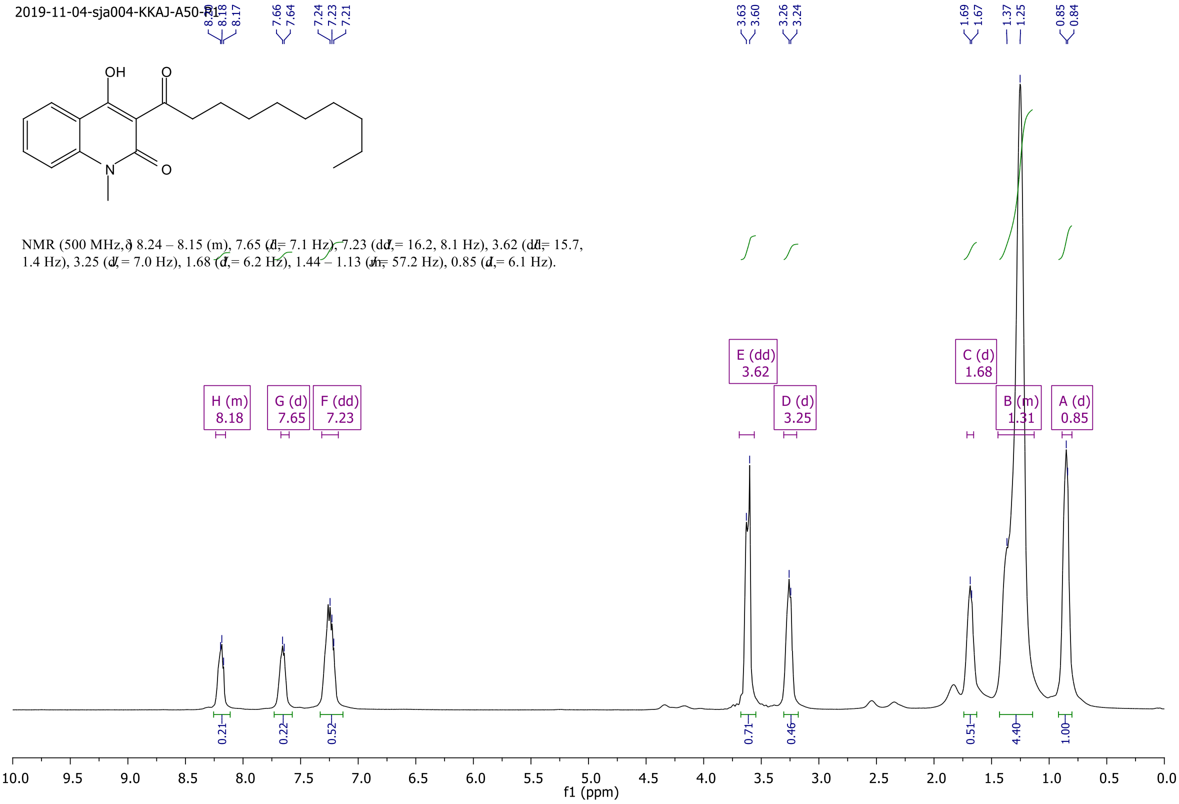

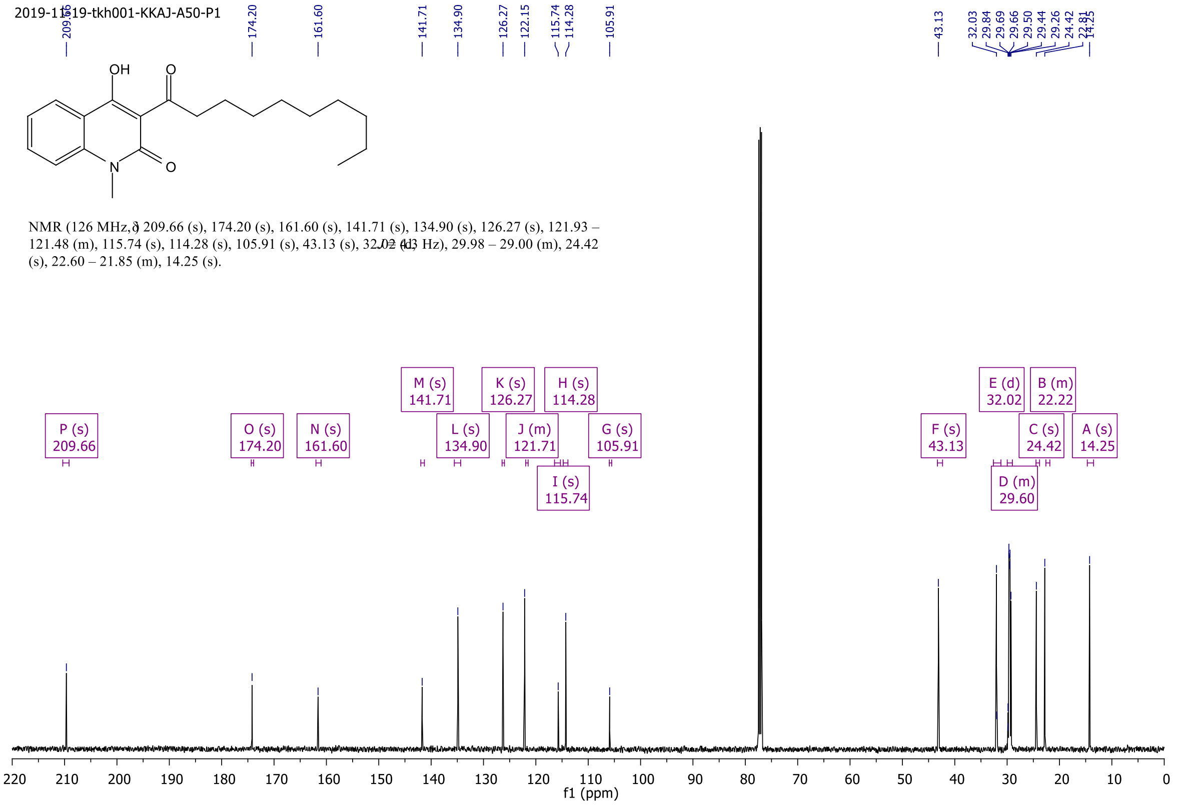

BIORESOURCES RESEARCH UNIT

High resolution report

Analysis Name	D:\Data\customer\TK 3h.d	Acquisition Date	6/1/2020 3:03:32 PM
Method	NaFormate_pos.m	Operator	Sutichai Ext: 3560
Sample Name	TK 3h	Instrument	micrOTOF Bruker
		Calibrate by	Sodium Formate

Acquisition Parameter

Source Type	ESI	Ion Polarity	Positive	Set Nebulizer	0.3 Bar
Focus	Not active			Set Dry Heater	180 ?C
Scan Begin	100 m/z	Set Capillary	4500 V	Set Dry Gas	4.0 l/min
Scan End	2000 m/z	Set End Plate Offset	-500 V	Set Divert Valve	Source

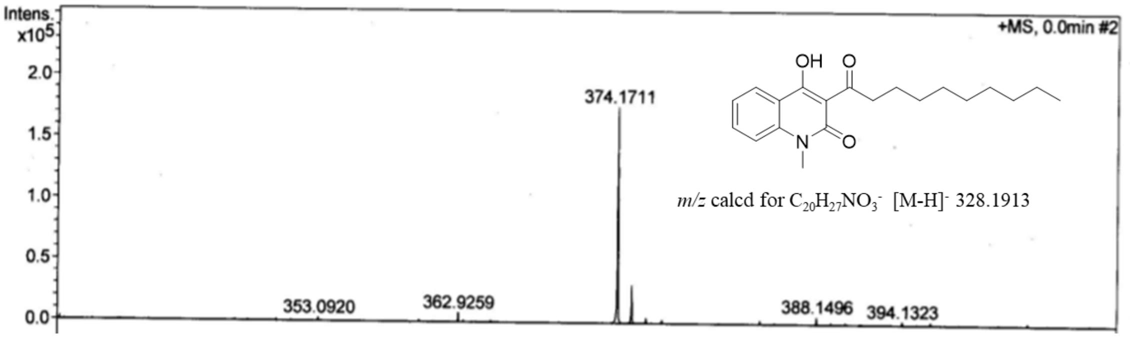

6-Chloro-3-decanoyl-4-hydroxy-1-methylquinolin-2(1H)-one (**3i**)

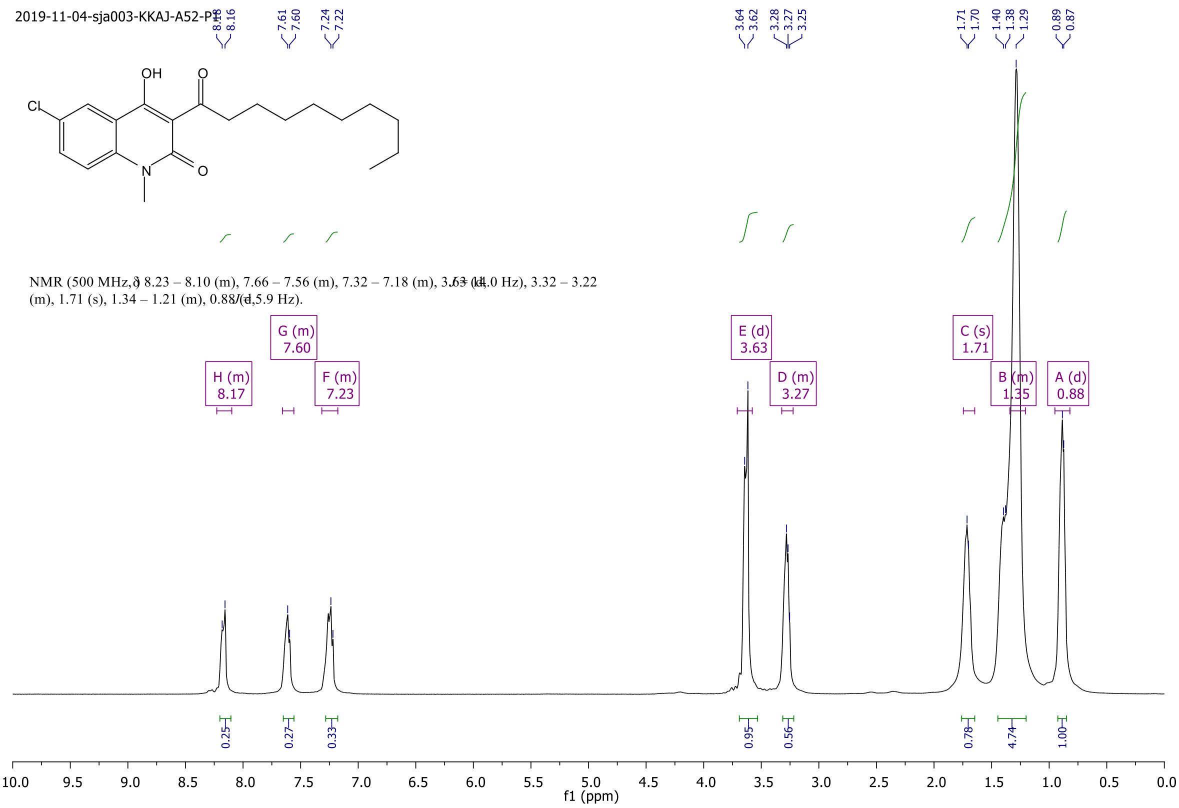

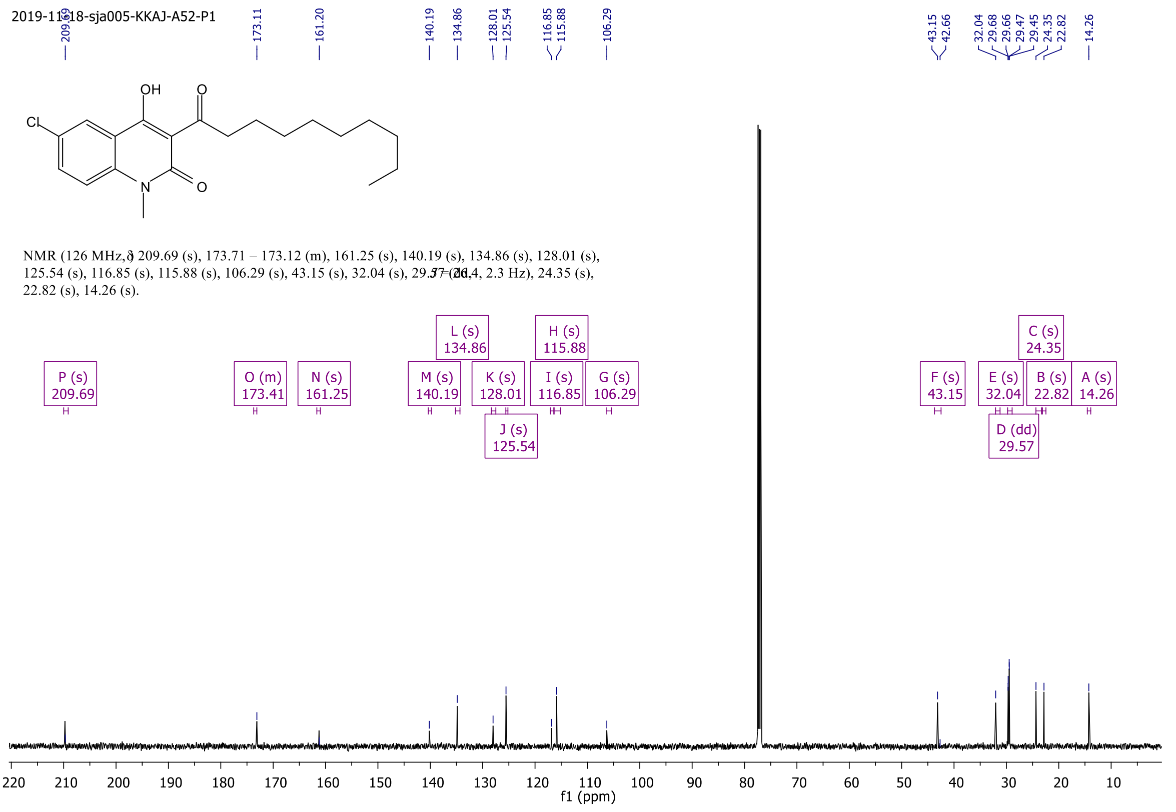

BIORESOURCES RESEARCH UNIT

High resolution report

Analysis Name	D:\Data\customer\TK 3i.d	Acquisition Date	6/1/2020 3:14:55 PM
Method	NaFormate_pos.m	Operator	Sutichai Ext: 3560
Sample Name	TK 3i	Instrument	micrOTOF Bruker
		Calibrate by	Sodium Formate

Acquisition Parameter

Source Type	ESI	Ion Polarity	Positive	Set Nebulizer	0.3 Bar
Focus	Not active			Set Dry Heater	180 ?C
Scan Begin	100 m/z	Set Capillary	4500 V	Set Dry Gas	4.0 l/min
Scan End	2000 m/z	Set End Plate Offset	-500 V	Set Divert Valve	Source

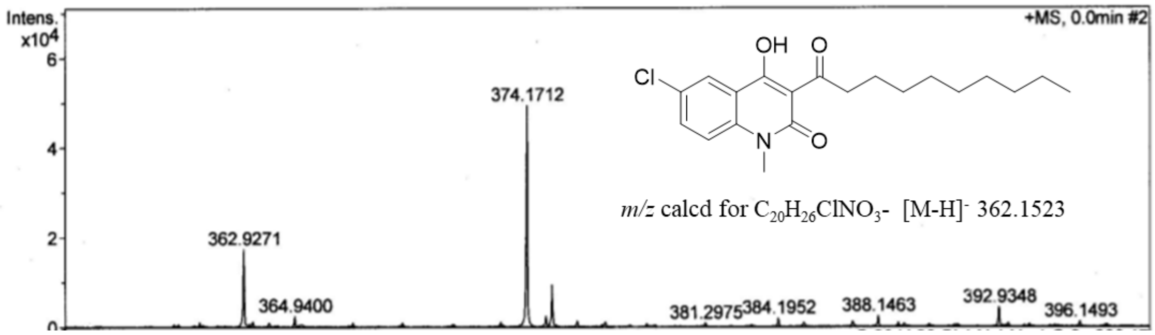

6-Bromo-3-decanoyl-4-hydroxy-1-methylquinolin-2(1H)-one (**3j**)

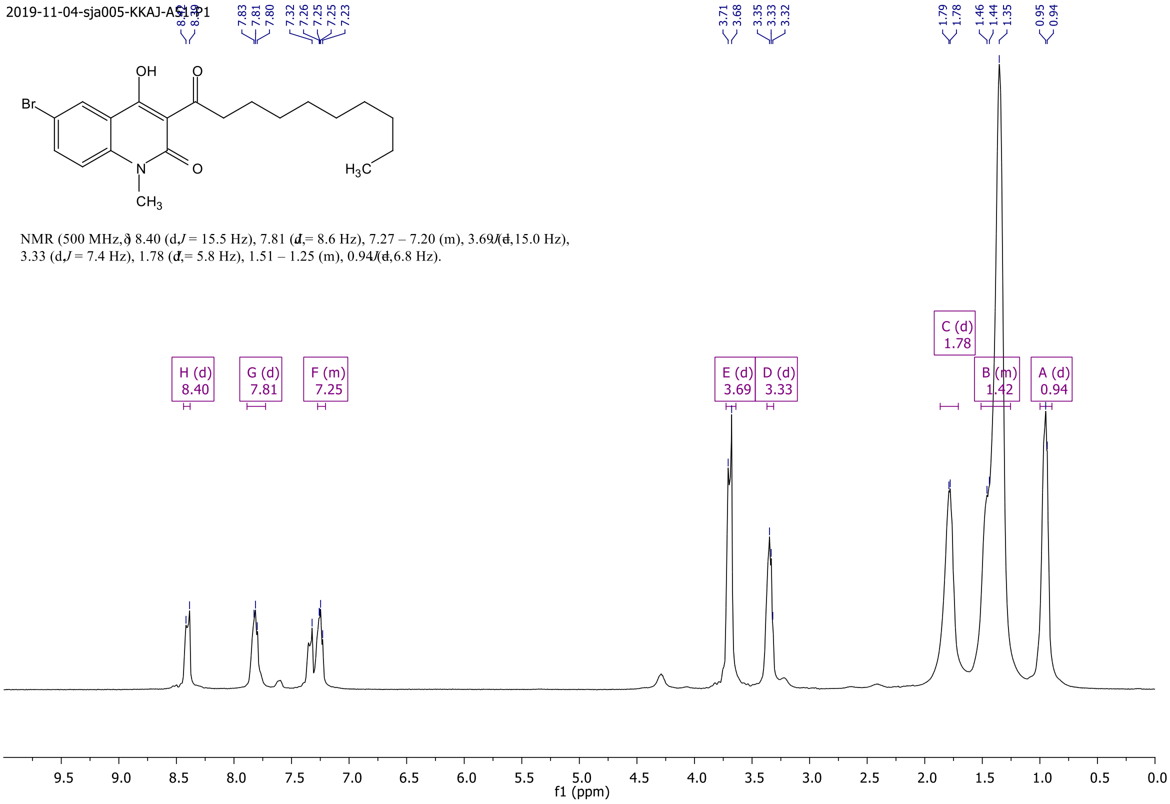

NMR (500 MHz, δ 8.40 (d, J = 15.5 Hz), 7.81 (d, = 8.6 Hz), 7.27 – 7.20 (m), 3.69 (d, 15.0 Hz), 3.33 (d, J = 7.4 Hz), 1.78 (d, = 5.8 Hz), 1.51 – 1.25 (m), 0.94 (d, 6.8 Hz).

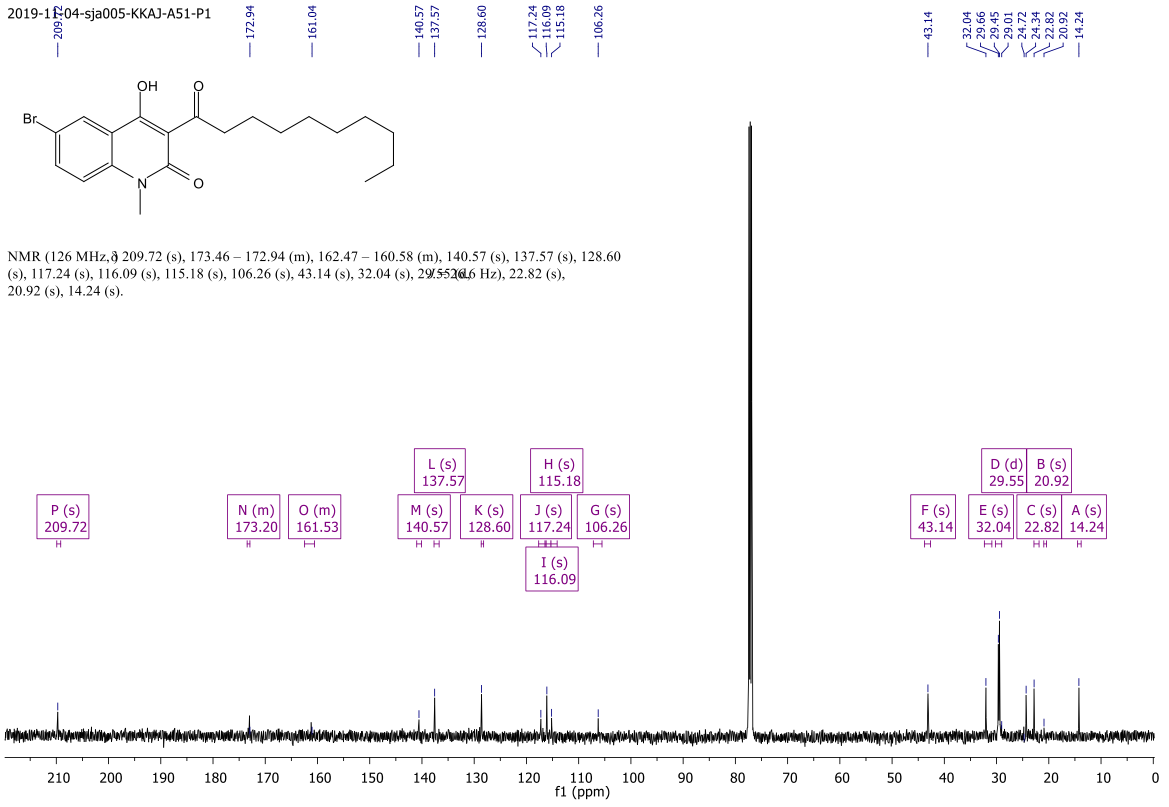

NMR (126 MHz, δ 209.72 (s), 173.46 – 172.94 (m), 162.47 – 160.58 (m), 140.57 (s), 137.57 (s), 128.60 (s), 117.24 (s), 116.09 (s), 115.18 (s), 106.26 (s), 43.14 (s), 32.04 (s), 29.55 (d, 6 Hz), 22.82 (s), 20.92 (s), 14.24 (s).

BIORESOURCES RESEARCH UNIT

High resolution report

		Acquisition Date	6/1/2020 3:26:20 PM	
Analysis Name	D:\Data\customer\TK 3j.d			
Method	NaFormate_pos.m	Operator	Sutichai	Ext: 3560
Sample Name	TK 3j	Instrument	micrOTOF	Bruker
		Calibrate by	Sodium Formate	

Acquisition Parameter

Source Type	ESI	Ion Polarity	Positive	Set Nebulizer	0.3 Bar
Focus	Not active			Set Dry Heater	180 ?C
Scan Begin	100 m/z	Set Capillary	4500 V	Set Dry Gas	4.0 l/min
Scan End	2000 m/z	Set End Plate Offset	-500 V	Set Divert Valve	Source

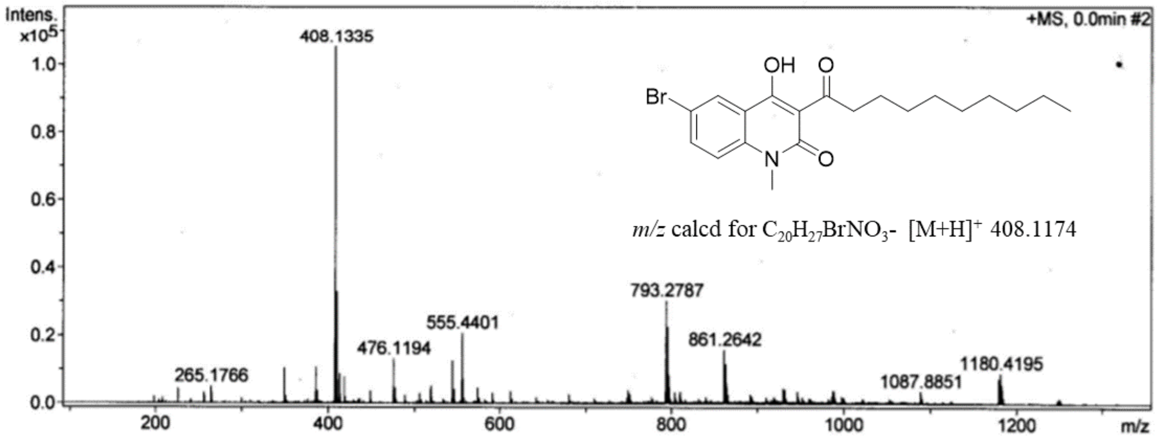

2. Antimicrobial Activity and Structure-Activity Relationship

2.1 The resazurin microdilution assay to determine MICs of the synthesized quinolones against *S. aureus*.

The synthesized compounds (**3a**, **3b**, **3c**, **3d**, **3e**, **3f**, **3g**, **3h**, **3i**, **3j**, and **4d**) were incubated with *S. aureus* (ATCC 6538P) at the final compound concentrations of 1000, 500, 250, 125, 62.5, and 31.25 µg/mL. The 4% DMSO in MHB, 4% DMSO in MHB plus *S. aureus*, and ciprofloxacin were used as experimental controls. MIC was defined as a minimum concentration of agents that inhibited the growth of bacteria observed by remaining blue color of resazurin.

The MICs of compound **3i** (*) and **3j** (**) were unclear. However, the **3i** and **3j** seemed to display partial inhibitory effect against *S. aureus* at the concentration between 125-1000 µg/mL and 125-500 µg/mL, respectively.

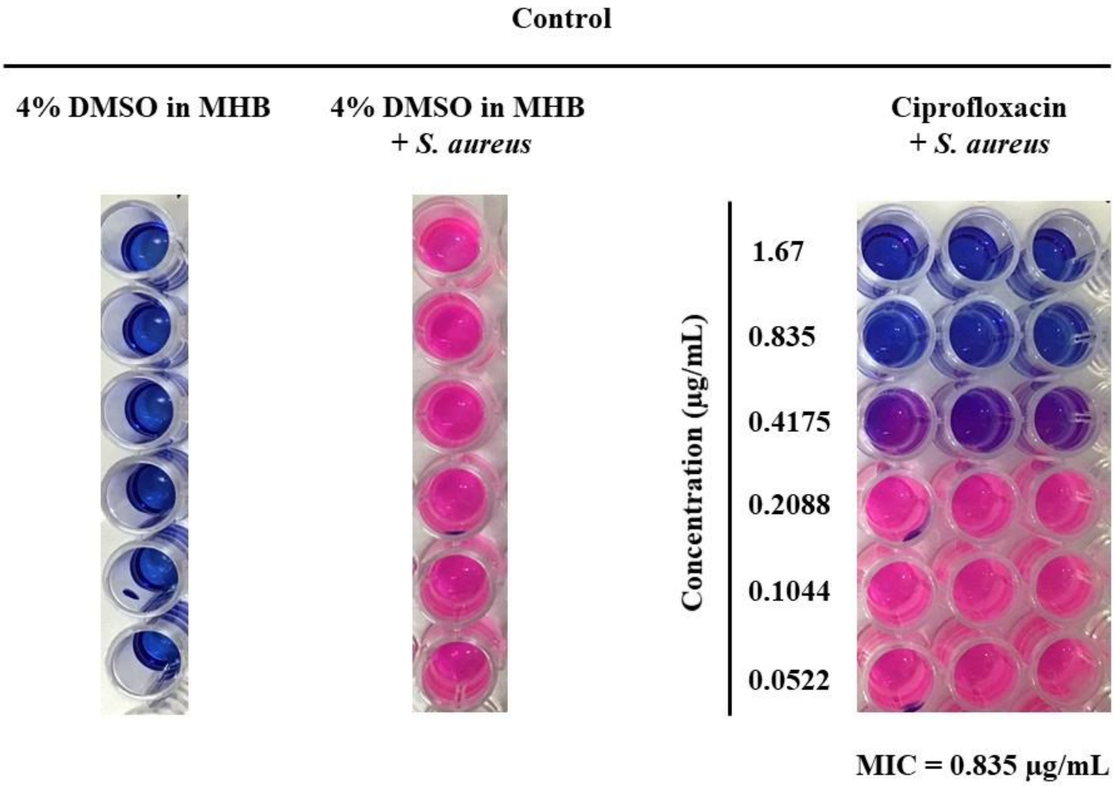

Figure S1. MIC determination of Ciprofloxacin against *S. aureus.*

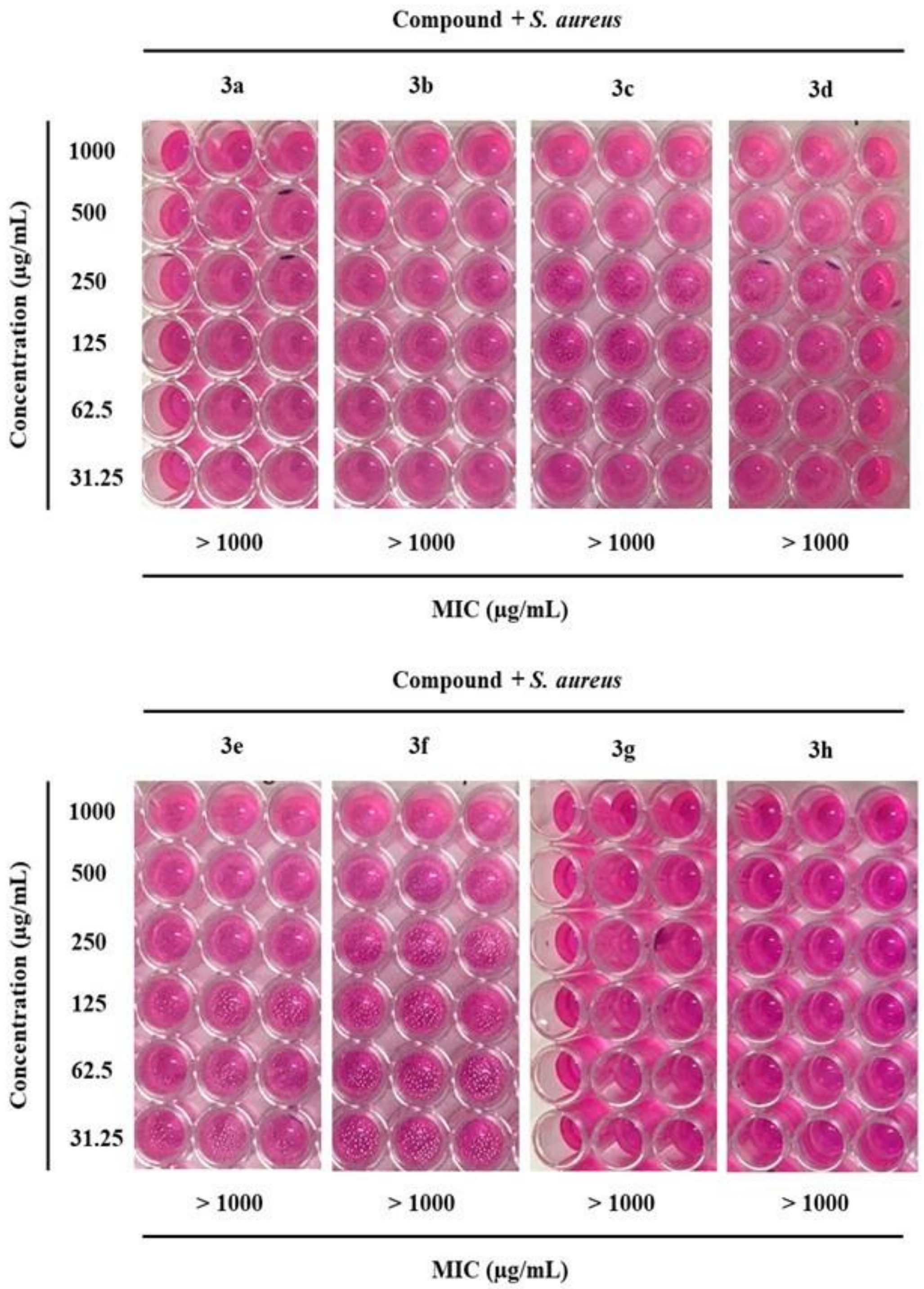

Figure S2. MIC determination of **3a**–**3h** against *S. aureus.*

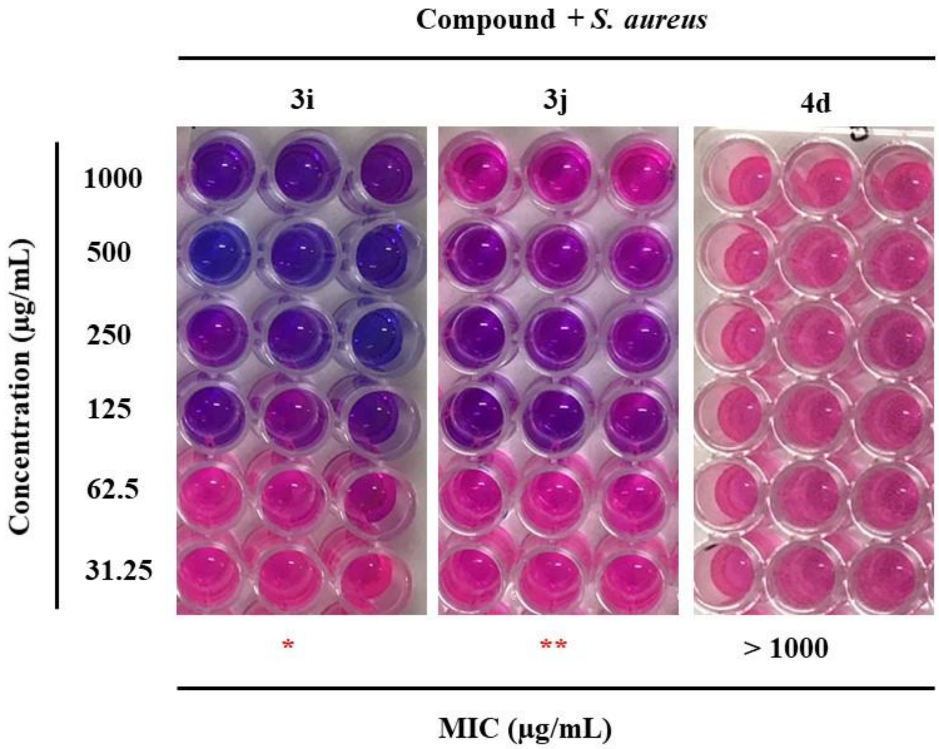

Figure S3. MIC determination of **3i**, **3j** and **4d** against *S. aureus.*

2.2 The MBC results of 3i and 3j against *S. aureus*.

Although 3i and 3j showed inhibitory activity against *S. aureus*, both compounds failed to kill the bacteria confirmed by the presence of bacterial colonies on nutrient agar plates.

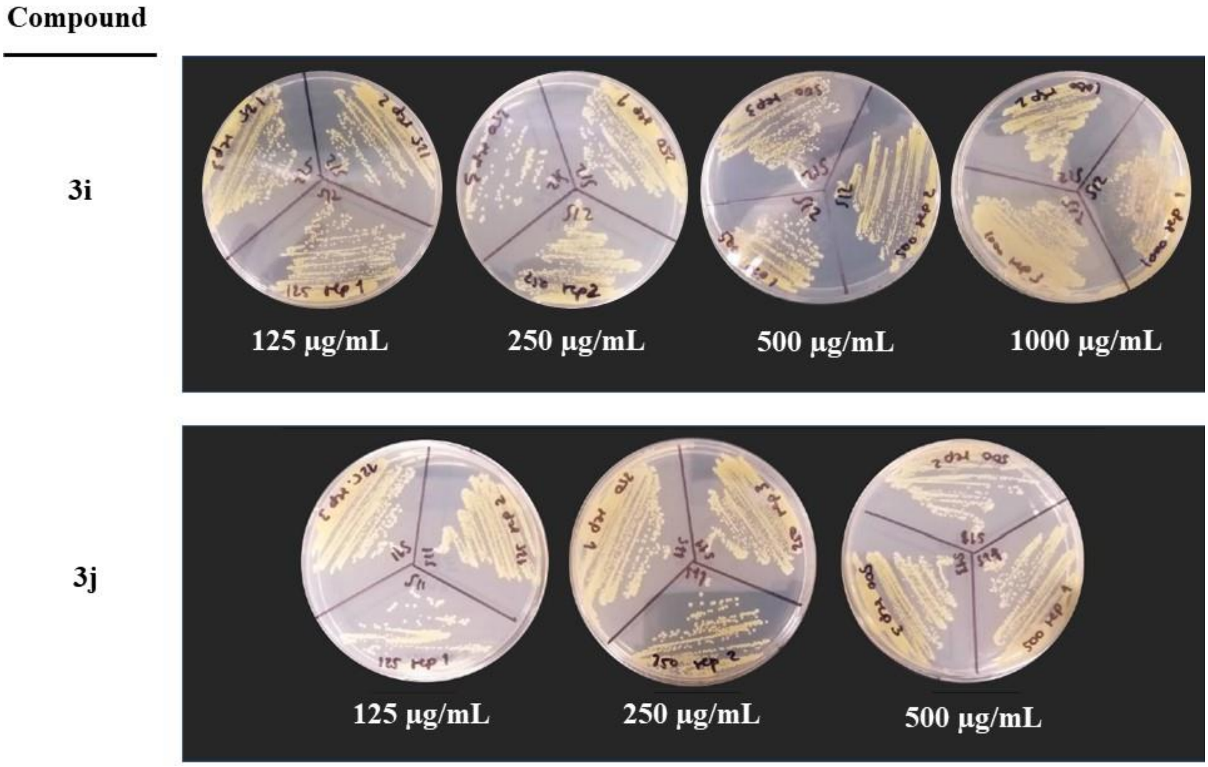

Figure S4. MBC determination of **3i** and **3j** against *S. aureus.*

2.3 The resazurin microdilution assay to determine MICs of the synthesized quinolones against *E. coli*.

The synthesized compounds (**3a**, **3b**, **3c**, **3d**, **3e**, **3f**, **3g**, **3h**, **3i**, **3j**, and **4d**) were incubated with *E. coli* (ATCC 25922) at the final compound concentrations of 1000, 500, 250, 125, 62.5, and 31.25 µg/mL. The 4% DMSO in MHB, 4% DMSO in MHB plus *E. coli*, and ciprofloxacin were used as experimental controls. MIC was defined as a minimum concentration of agents that inhibited the growth of bacteria observed by remaining blue color of resazurin.

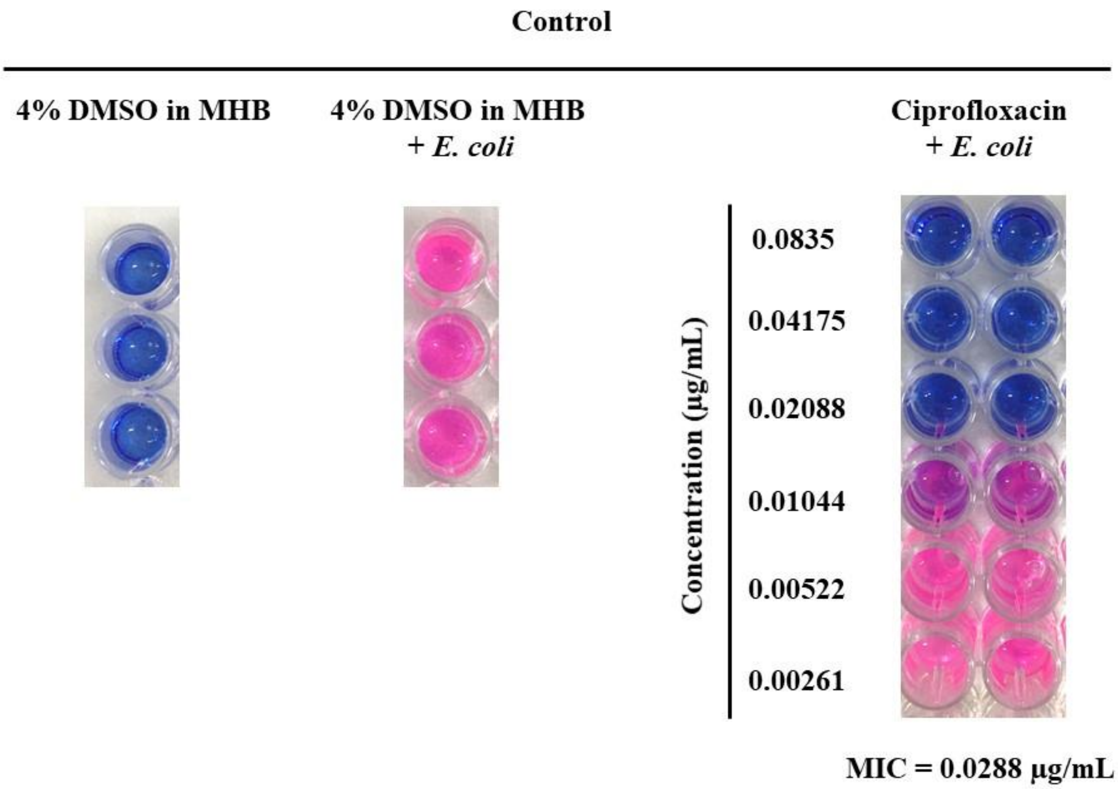

Figure S5. MIC determination of Ciprofloxacin against *E. coli.*

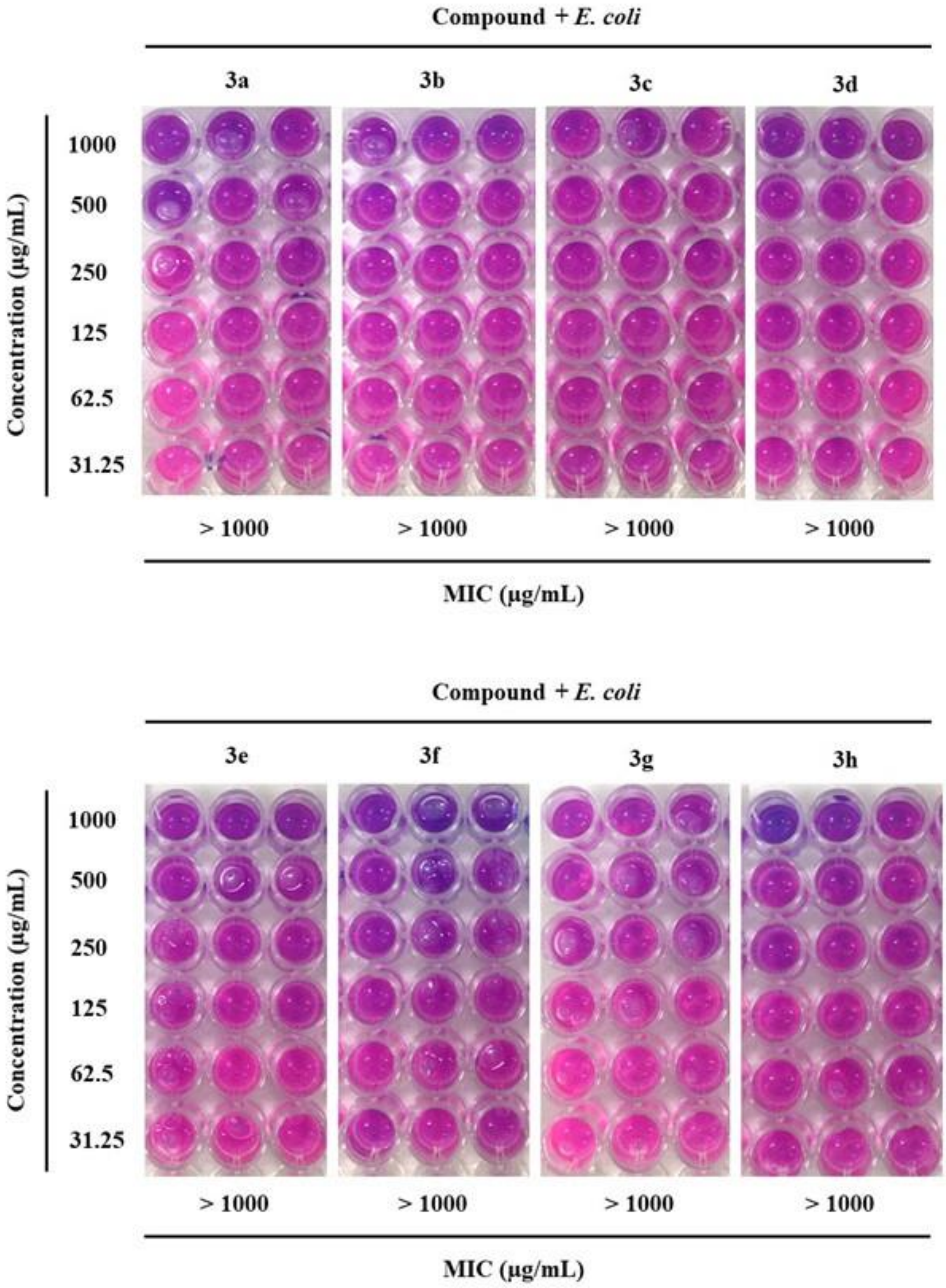

Figure S6. MIC determination of **3a**–**3h** Ciprofloxacin against *E. coli.*

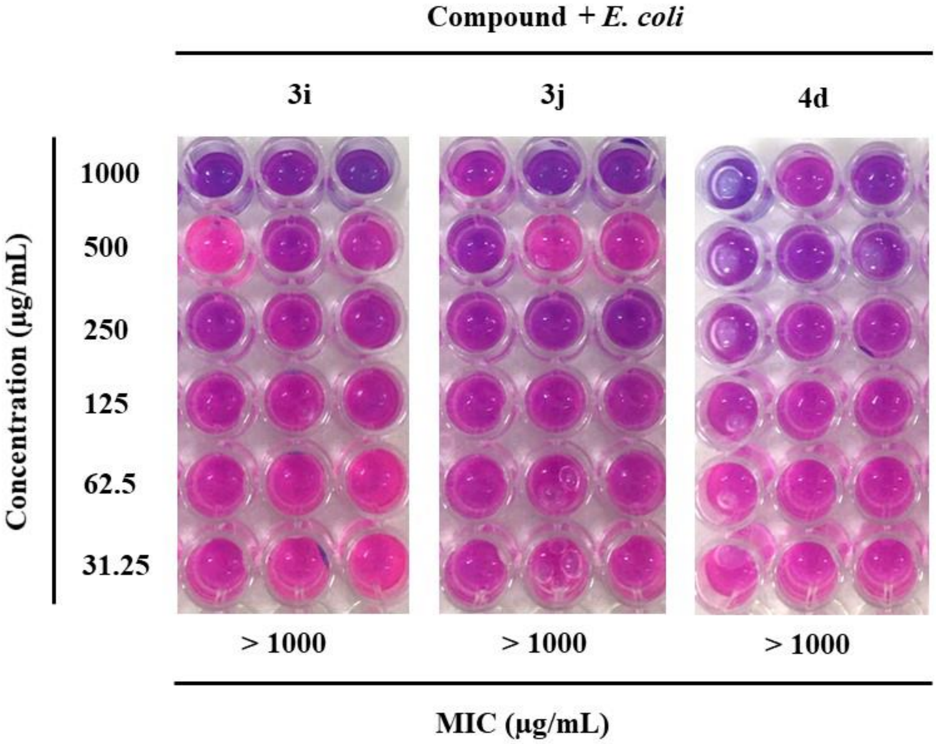

Figure S7. MIC determination of **3i**, **3j** and **4d** against *E. coli.*
